# Complex Evolutionary History of *Mboumar*, a *Mariner* Element Widely Represented in Ant Genomes

**DOI:** 10.1038/s41598-020-59422-4

**Published:** 2020-02-13

**Authors:** Olivia Sanllorente, Jesús Vela, Pablo Mora, Areli Ruiz-Mena, María Isabel Torres, Pedro Lorite, Teresa Palomeque

**Affiliations:** Departamento de Biología Experimental, Área de Genética, Universidad de Jaén, 23071 Jaén, Spain

**Keywords:** Evolution, Genetics

## Abstract

*Mboumar-9* is an active *mariner*-transposable element previously isolated in the ant *Messor bouvieri*. In this work, a *mariner*-like element, *Mboumar*, isolated from 22 species of ants, is analyzed. These species belong to nine different subfamilies, including Leptanillinae, the most primitive ant subfamily, and Myrmicinae and Formicidae, the most derived ones. Consequently, *Mboumar*-like elements seem to be well-represented in ant genomes. The phylogenetic tree drawn for *mariner* elements is highly inconsistent with the phylogeny of host ants, with almost identical elements found in clearly distant species and, on the contrary, more variable elements in closely related species. The inconsistency between the two phylogenetic trees indicates that these transposable elements have evolved independently from the speciation events of the ants that host them. Besides, we found closer genetic relationships among elements than among their host ants. We also found potential coding copies with an uninterrupted open reading frame of 345 aa in 11 species. The putative transposase codified by them showed a high sequence identity with the active Mboumar-9 transposase. The results of selection tests suggest the intervention of purifying selection in the evolution of these elements. Overall, our study suggests a complex evolutionary history of the *Mboumar*-like *mariner* in ants, with important participation of horizontal transfer events. We also suggest that the evolutionary dynamics of *Mboumar*-like elements can be influenced by the genetic system of their host ants, which are eusocial insects with a haplodiploid genetic system.

## Introduction

Transposable elements (TEs) are very frequent in the genomes of most organisms^1,2^. They are capable of moving from one chromosomal site to another within a genome. Eukaryotic TEs have been classified into two classes, according to their molecular mechanism of transposition^2^. Class I elements (or retrotransposons) transpose through an RNA intermediate, which is reverse transcribed by a “copy and paste mechanism”. Class II elements (or DNA transposons) move within a genome by an excision-insertion mechanism (“cut and paste mechanism”) by using the enzyme transposase. *Mariner*–like elements (MLEs) are Class II elements belonging to the *Tc1-mariner* family, a member of the *IS630-Tc1-mariner* superfamily, which is present in almost all living organism groups, including eukaryotic and prokaryotic species^3–6^. MLEs display a simple structure with a size of approximately 1300 bp in length flanked by two inverted terminal repeats (ITRs) and containing a single gene which encodes a transposase. MLE transposases have two domains: the N-terminal ITR-binding domain containing helix-turn-helix (HTH) motifs, and a C-terminal catalytic domain that contains the conserved and characteristic motif D,D(34)D.

Several authors^7^ have differentiated three stages in the so-called *mariner* life cycle that may be applicable and general for all TEs^8^. The cycle begins with the invasion of a new host by horizontal transfer (HT), followed by its proliferation in the host genome. The third stage includes vertical inactivation as a result of mutation accumulations. Over time, the MLEs become nonfunctional, and the divergence between the different copies increases. Genetic drift and/or natural selection processes and/or stochastic loss might lead to the loss of active or inactive MLE sequences from a genome. Horizontal transfer events allow the invasion of new genomes and prevent loss of the transposon by the genetic drift process^9^. Consequently, HT events are the key step in the TE life cycle. This step of HT is under selection since the transposition and invasion of a new genome requires an active element with full enzymatic activity^10,11^. However, when the elements are already installed within a host, they can evolve under a pattern of neutral evolution. In the absence of selection, the mutations may accumulate, explaining the vertical inactivation. The horizontal transfer of a *P* element between *Drosophila melanogaster* and *Drosophila willistoni* was the first report concerning HT events^12^. Cross-mobilization events have also been described in *Drosophila*, concretely in the *Drosophila mauritiana* genome, between a full-length inactive *peach* copy and the transposase of *Mos1*^13^ and subsequently, in other TEs^14,15^.

Three signals could suggest the existence of HT: the discontinuous presence of a TE in a group of taxa, unusually high similarity among TEs from genetically distant host species, and incongruence between TEs and host phylogenies. However, these signals can also be explained by a vertical-transmission process if other processes are also involved as different evolutionary rates between species, stochastic losses, or ancestral polymorphisms^9,16,17^. For the above reasons, other methods are also applied. It is now accepted that one of the best methods for inferring HT is the comparison of nucleotide divergences at synonymous sites (*K*_*S*_) in both TEs and the hosts’ nuclear genes. Under HT, there is lower divergence in TE sequences than in host sequences^18–20^.

In ant genomes, four *mariner* elements have been described, three of which belong to the *mauritiana* subfamily of *Tc1*/*mariner* transposons, concretely, *Myrmar* in *Myrmica ruginodis*^21^, *Sinvmar* in *Solenopsis invicta*^22^, and *Mboumar in Messor bouvieri*^23^*. Mboumar-9* is an active TE^24^. The fourth element, *Tnigmar-Az*, which belongs to the *irritans* subfamily and is also included in the *Tc1*/*mariner* superfamily, has been isolated from *Tapinoma nigerrimum*. This *mariner* has also been found in the genome of several ant species^25,26^. The existence of HT events has been reported for the *Tnigmar-Az mariner*^25,26^.

In this work, *Mboumar*-like elements isolated from 22 ant genomes are analyzed. The ants belong to nine different subfamilies, and consequently, *Mboumar*-like elements seem to be well represented in the ant genomes. We suggest a complex evolutionary history of this element with the strong participation of horizontal transfer events.

## Results

### Isolation of *Mboumar*-like elements, and sequence and phylogenetic analysis

*Mboumar* elements isolated from *Messor bouvieri* were found to be inserted in a miniature inverted-repeat transposable element (MITE)^23^. PCR amplification assays were performed using a single primer (MEBOTRA) based on the terminal repeats (ITRs) of this MITE. However, the majority of PCR amplification assays were performed using the primer ITR-MAR based on the ITR sequences of *Mboumar mariner*^25^, as explained in the Material and Methods section. In 20 ant species, PCR bands of about 1300 bp (potential complete *mariner* elements) were subsequently cloned and sequenced. These species belong to nine ant subfamilies. The sequences were deposited in the EMBL sequence database (Table S1). Parts of the analyzed sequences (46%) were 1287 bp in length; the remaining sequences show small differences in length as a consequence of indels (Fig. S1). In this study, previously obtained *mariner* elements from *M. bouvieri* (*Mboumar*) and *T. ibericum* have also been included (formerly named the *T. nigerrimum* and *Tnigmar-Mb* element)^23,25^. In the conditions used, PCR amplification was not obtained in the other 26 ant species. These species belong to four different ant subfamilies: Formicinae, Myrmicinae, Dolichoderinae, and Myrmeciinae. However, for other species of these subfamilies, we could amplify *Mboumar*-like *mariner* elements, revealing a discontinuous distribution of these TEs in ants (Fig. S3). In fact, we found several ant genera, such as *Aphaenogaster*, *Camponotus*, or *Myrmecia*, including species with and without *Mboumar* elements.

**Figure 1:**
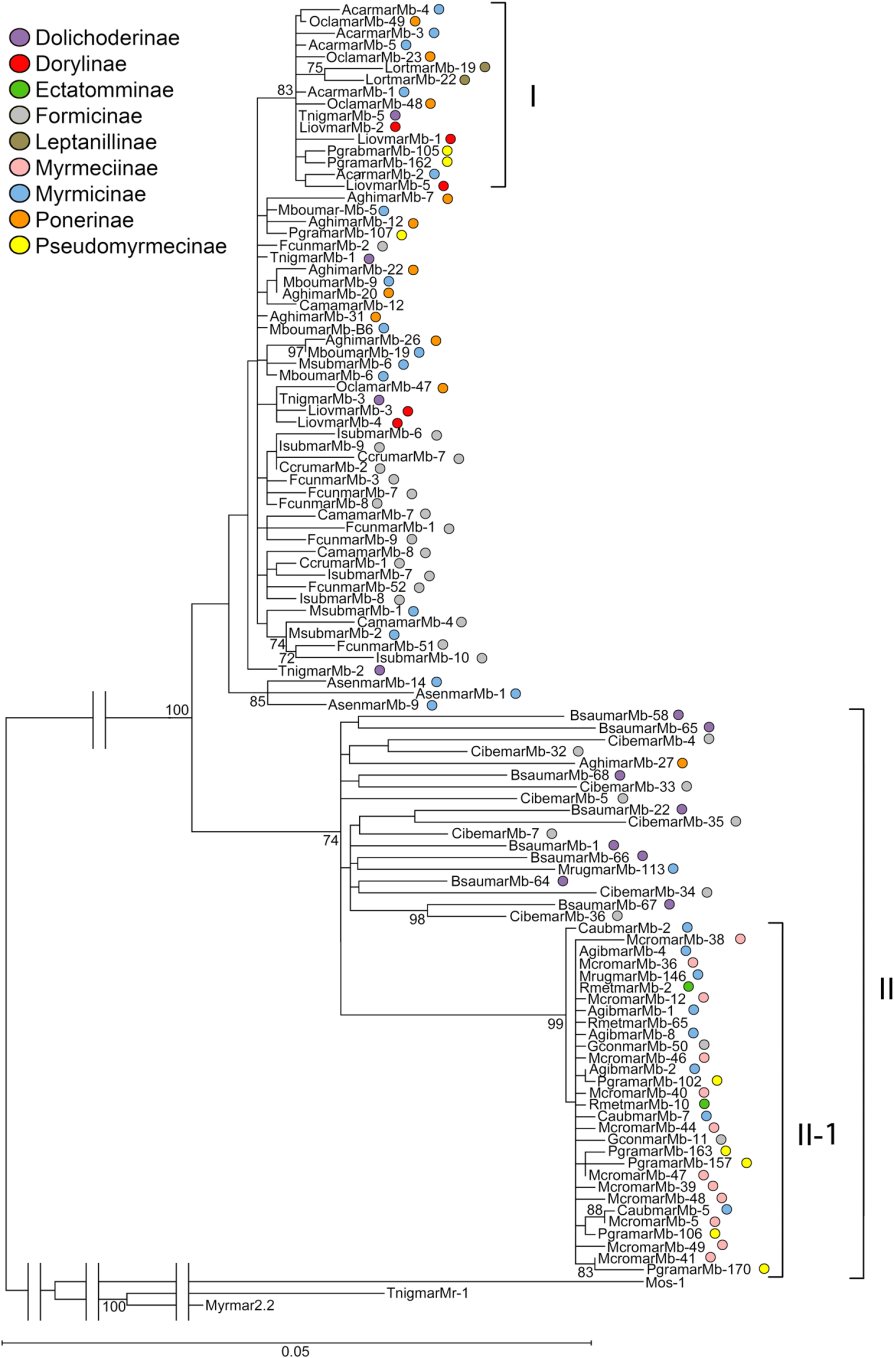
Maximum-likelihood analysis of the nucleotide *Mboumar-*like *mariner* sequences. Numbers indicate the bootstrap values over 1000 replications. Only bootstrap support values greater than 70% are indicated.

The evolutionary divergence between *mariner* sequences within each one of the 22 analyzed species shows very low values (0.001 to 0.036) (Table S2). Similarly, the evolutionary divergences between elements isolated from different species also show very low values (0.001 to 0.050) (Table S3). Accordingly, the average evolutionary divergence over all studied sequence pairs was also very low (0.028 ± 0.003). For some species, the number of sequences analyzed is low, but the data suggest that the obtained sequences are representative of the *Mboumar*-like variability within each species. For example, in *M. croslandi*, 12 sequences have been sequenced and the intraspecific variability is one of the lowest values (0.003) (Table S2), and all of them are included in Subclade II-1 (Fig. 1). For several species, the sequences were amplified in different PCRs obtaining similar results. Even sequences obtained in the same species using MEBOTRA or ITR-MAR primers were similar and belonged to the same group (Fig. S1).

A phylogenetic analysis was carried out using the 105 analyzed *mariner* sequences isolated in 22 species. Among the sequences, a group of 45 sequences (isolated in 10 species) are grouped in a clade (Clade II) with relatively low bootstrap support (Fig. 1). Inside this clade, a highly supported clade, including 27 sequences, is differentiated (Subclade II-1). Among the remaining 60 sequences isolated in 14 species, only 16 sequences cluster together in a well-supported clade (Clade I). Therefore, we found 44 non-clustered sequences. Consequently, three groups of sequences could be defined: Clade I, Subclade II-1, and remaining sequences of Clade II. The phylogenetic tree shows that the elements hosted by each ant species are not clustered together. The only exceptions are *Asenmar-Mb* sequences, isolated from *Aphaenogaster senilis*, which cluster together. In addition, the branches are very short, as expected due to the low evolutionary divergences among *mariners*. For several species, some of the *mariner* sequences are included in different groups, as we will discuss later (Table 1). The nucleotide divergences between elements included in the same group are smaller than the divergences between elements of different groups (Table S4). The fixed differences between them are also shown in Table S4.

**Table 1:** Subfamily, ant species hosts, and name of elements hosted by them. Potentially active copies are marked with an asterisk.

Subfamily	Species	Name of element
**Clade I**		
Myrmicinae	*Aphaenogaster cardenai*	*Acarmar-Mb-*1, −2, −3, −4 and, −5
Dolichoderinae	*Tapinoma ibericum*	*Tnigmar-Mb-5*
Pseudomyrmecinae	*Pseudomyrmex gracilis*	Pgramar-*Mb*-105 and −162
Dorylinae	*Lioponera iovis*	*Liovmar-Mb-*1, −2 and −5
Ponerinae	*Odontomachus clarus*	*Oclamar-Mb*-23, −48 and −49
Leptanillinae	*Leptanilla ortunoi*	*Lortmar-Mb*-19 and −22
**Clade II, Subclade II-1**		
Myrmicinae	*Aphaenogaster gibbosa*	*Agibmar-Mb-*1 and −8
	*Crematogaster auberti*	*Caubmar-Mb-*2, −5 and −7
	*Myrmica ruginodis*	*Mrugmar-Mb*-146
Ectatomminae	*Gnamptogenys continua*	*Gconmar-Mb-*11 and −50
	*Rhytidoponera metallica*	*Rmetmar-Mb*-2 and 10
Pseudomyrmecinae	*Pseudomyrmex gracilis*	*Pgramar-Mb-*102, −106, −157, −163 and −170
Myrmeciinae	*Myrmecia croslandi*	*Mcromar-Mb-*5, −12, −36, −38, −39, −40, −41, −44, −46, −47, −48 and −49
**Clade II, remaining sequences**		
Myrmicinae	*Myrmica ruginodis*	*Mrugmar-Mb*-113
Formicinae	*Cataglyphis iberica*	*Cibemar-Mb*-4, −5, −7, −32, −33, −34, −35 and −36
Dolichoderinae	*Bothriomyrmex saundersi*	*Bsaumar-Mb*-1, −22, −58, −64, −65, −66, −67 and −68
Ponerinae	*Anochetus ghilliani*	*Aghimar-Mb*-27*
**Non-clustered sequences**		
Myrmicinae	*Aphaenogaster senilis*	*Asenmar-Mb*-9*,−12* and 14
	*Messor bouvieri*	*Mboumar-Mb-*5, −6*, −9*, −19* and B6*
	*Monomorium subopacum*	*Msubmar-Mb-*1*, −2* and −6
Formicinae	*Camponotus amaurus*	*Camamar-Mb*-4, −7, − 8* and 12*
	*Camponotus cruentatus*	*Ccrumar-Mb*-1*, −2 and 7
	*Formica cunicularia*	*Fcunmar-Mb*-1, −2*, −3*, −7*, −8*, −9, −51* and −52*
	*Iberoformica subrufa*	*Isubmar-Mb*-6, −7*, −8*, −9 and −10
Dolichoderinae	*Tapinoma ibericum*	*Tnigmar-Mb -*1*, −2* and −3*
Pseudomyrmecinae	*Pseudomyrmex gracilis*	*Pgramar-Mb*-107
Dorylinae	*Lioponera iovis*	*Liovmar-Mb*-3* and −4*
Ponerinae	*Anochetus ghillianii*	*Aghimar-Mb*-7, −12, −20*, −22*, −26* and −31
	*Odontomachus clarus*	*Oclamar-Mb*-47*

According to our results, there is no relationship between the *mariner* groups and the subfamily of the host ant. Within each group, there are *mariners* from ant species from several subfamilies. Regardless of the subfamily to which they belong, generally all sequences isolated from one ant species are included in one of the groups (Fig. 1, Table 1). However, there are some exceptions; three species present sequences included in Clade I and in the non-clustered sequences (*Tapinoma ibericum*, *Lioponera iovis*, and *Odontomachus clarus*). These three species belong to three different subfamilies. The sequences isolated in *Anochetus ghilianii* are included in Clade-II and in the non-clustered sequences. The sequences isolated from *Myrmica ruginoides* are included in Clade II and in Subclade II-1. The most notable exception is found in *Pseudomyrmex gracilis*, with sequences included in Clade I, Subclade II-1, and in the non-clustered sequences.

Finally, within each group, there are several shared mutations among *mariner* sequences that are not present in the sequences of other groups (Fig. S1 and Table S4). For example, positions 33–36 (in relation to the consensus sequence) are GTAA in all sequences of Clade-II, but CCGG in the remaining sequences. The transition C to T (position 566) in Clade-I and the seven bp deletion (position 435–441) in Subclade II-1 generate a stop codon in all sequences. The greatest fixed nucleotide differences (46) are found between Clade I and the sequences of Subclade II-1.

### Study of putative transposases

Among the analyzed *Mboumar*-*mariner* copies, 29 of them could encode a putative transposase with 345 aa. These sequences belong to 11 different species included in five evolutionarily very different subfamilies (Table 1). There is an extremely high amino acid identity (95%–100%) between Mboumar transposase and the putative proteins that could encode these sequences (Fig. S2). The catalytic D,D(34)D motif in the C-terminal domain of active transposases is conserved in 23 of the 29 sequences. In Mboumar-B6, the first aspartic acid (D) has been replaced by glycine (G) and in the other four sequences, the third aspartic acid (D) has been replaced by tyrosine (Y). The bipartite nuclear location signal (NLS); the two highly conserved amino-acid motifs, WVPHEL and YSPDLAP(I/S/T); and other features of active *mariners*, are also wholly or partially conserved. Interestingly, all protein sequences except for Aghimar-Mb-20 (protein with 100% identity with Mboumar-9) and Aghimar-Mb-22 showed the basic amino acid lysine (K) at position 24. Mboumar-9 and Mos1 transposases^27^ showed only 68% identical amino acid sequences and in this position, Mboumar-9 shows the basic amino acid arginine (R) and Mos1 shows the neutral polar amino acid threonine (T), and both are active *mariners*. Since lysine and arginine have similar physical properties, it is probable that this amino acid replacement does not cause a significant change in the transposase activity.

Furthermore, the corresponding nucleotide sequences show a putative TATA box (at nucleotide position 55–64) and a polyadenylation signal (position 1204–1213) in 5′ and 3′untranslated regions. Among the 29 potentially active transposases, there are only two sequences that show a punctual mutation in the TATA box (*Aghimar-Mb*-27 and *Fcunmar-Mb*-51) (Fig. S1).

### Study of selection

The rates of synonymous (d_S_) and non-synonymous (d_N_) substitutions have been calculated in the coding region of all *mariner* sequences. Ratios close to 1 indicate neutral sequence evolution, ratios lower than 1 indicate purifying or negative selection, and ratios greater than 1 reflect positive selection. The average of d_N_ and d_S_ over all sequences was 0.0197 and 0.0434, respectively. Therefore, the overall value of d_N_/d_S_ is 0.454. We also performed codon-based Z-tests of selection. The codon-based test of purifying selection shows a probability (P) of 0.003 of rejecting the null hypothesis of no selection (d_N_ = d_S_) in favor of the alternative hypothesis (d_N_ < d_S_). Values of P lower than 0.05 are considered significant at the 5% level, according to MEGA software. Other authors, using other statistical tests to study selection, have also employed the same level of significance^28^. The statistical test (d_S_-d_N_) suggests purifying selection. The same study has been carried out within each group (Table S5). The results also suggest purifying selection within all considered groups.

### Evolutionary and comparative analysis between transposable elements and their host ants

**Figure 2:**
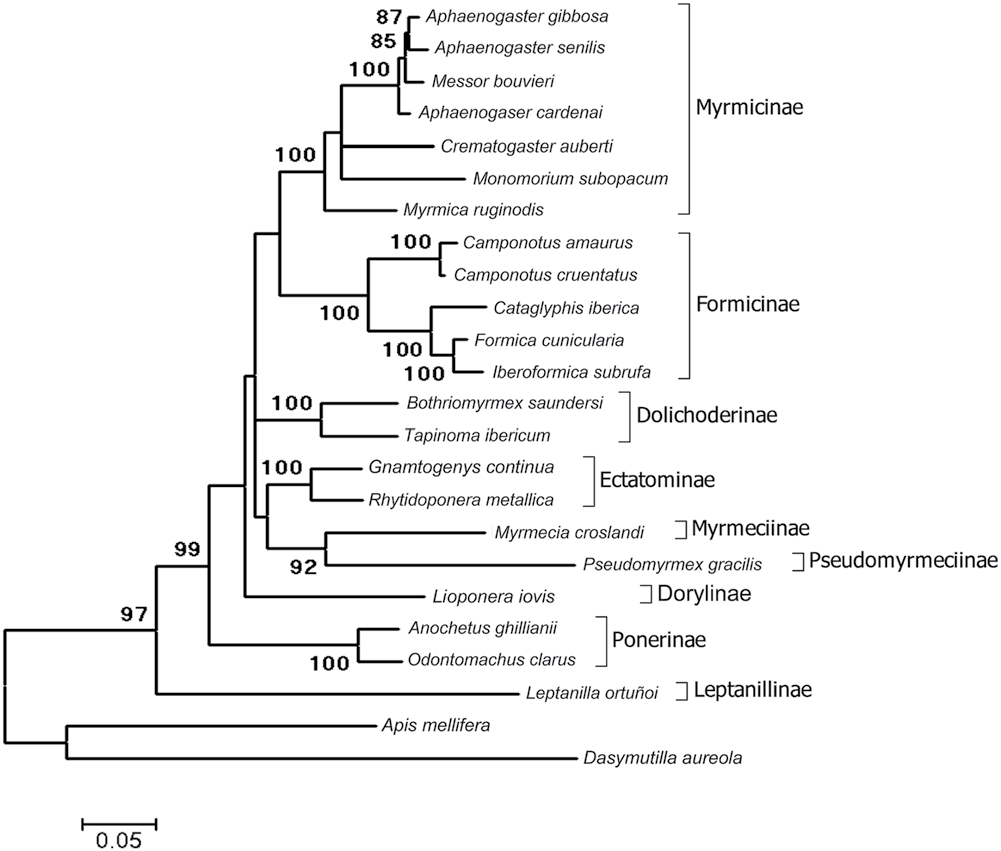
Reconstruction of the phylogenetic relationships among ant species. GenBank accession numbers of *wingless* (*wnt-1*), *abdominal-A* (*abdA*), and *long-wavelength rhodopsin* (*lwRh*) nucleotide sequences used for the ant phylogeny are given in Additional file 1: Table S1. Bootstrap support values greater than 80% are indicated next to each branch (1000 replicates).

The phylogenetic relationships among the host ants were inferred from the concatenated sequences of three single-copy nuclear loci: *wnt-1* (*wingless*), *abdA* (*abdominal-A*), and *lw-Rh* (*long-wavelength rhodopsin*) (Fig. 2). We estimated *K*_S_ (the average number of nucleotide substitutions at synonymous sites) to study the patterns of neutral divergence between host species and compared them, with similar estimates of *mariner* elements being isolated in each species. With this objective, we calculated pairwise *K*_S_ in the host species using the three single-copy nuclear loci (Table S6), as well as the corresponding *K*_S_ in *mariner* elements isolated in each species (Table S7). When the isolated sequences of a species were included in different groups, the corresponding estimates of *K*_S_ were estimated at the species level.

For all pairwise comparisons, the *K*_S_ among *mariner* elements is lower than the *K*_S_ among nuclear genes. This happens in species with potentially active *mariners* copies. For example, this is the case for *Camamar-Mb* and *Ccrumar-Mb* isolated from two species of the same genus, *Camponotus amaurus* and *C. cruentatus*, and between *Asenmar-Mb* and *Mboumar-Mb* isolated from *Aphaenogaster senilis* and *Messor bouvieri*, belonging to closely related genera. The same results are observed between *Fcunmar-Mb* and *Isubmar-Mb* isolated from *Formica cunicularia* and *Iberoformica subrufa*, which are sister genera according to molecular phylogenies^29^, and among phylogenetically distant genera, such as *Messor bouvieri, Tapinoma ibericum*, and *Anochetus ghillianii* (Fig. 2, Tables S6 and S7). Similar results are observed for species without potentially active copies. The absence of substitutions at synonymous sites in some *mariner* elements included in Subclade II-1 is remarkable (Table 1, Table S7). However, there were two exceptions. Specifically, the *K*_S_ between *Agibmar-Mb* (isolated from *Aphaenogaster gibbosa*) and *Acarmar–Mb* (isolated from *A. cardenai*) is greater than the *K*_S_ between the host species (0.10 and 0.07, respectively). Similarly, the *K*_S_ between *Agibmar-Mb* and *Asenmar-Mb*, isolated from *A. senilis*, is greater that the *K*_S_ between the host species (0.10 and 0.06, respectively). However, this result is logical as each *Aphaenogaster* species hosts a different type of *mariner* element included in different groups (Fig. 1, Table 1) and the divergence between them is similar to the divergence between the corresponding groups.

## Discussion

The *Mboumar* elements were first isolated in the ant *Messor bouvieri*^23^. Using primers based on the ITR sequences of *Mboumar*, similar sequences were isolated in *Tapinoma ibericum*^25^. Phylogenetic analysis with transposase amino acid sequences suggests that they belong to the *mauritiana* subfamily^25^ of the *IS630-Tc1-mariner* superfamily. A horizontal transfer event has been suggested to have occurred for this element about 2 million years ago^25^.

In the present study, *Mboumar-*like elements were isolated in another 20 ant species. One of these is *Leptanilla ortunoi*, from the subfamily Leptanillinae, the most primitive ant subfamily that separated from the others about 72–123 Mya^30,31^. Moreau *et al*.^30^ have suggested that the subfamily Leptanillinae can be considered to be a sister group to all other extant ants. These elements have also been isolated in ants of the subfamilies Ponerinae (Poneroid clade), Myrmicinae, and Formicidae (considered as the most derived), as well as in species from the subfamilies Ectatomminae, Dolichoderinae, Pseudomyrmecinae, Myrmecinae, and Dorylinae (all included in the Formicoid clade). The crown-group age estimates for Poneroid and Formicoid clades are between 80–152 Mya and 107–147 Mya, respectively^30,31^. Consequently, *Mboumar*-*like* elements seem to be widely represented in ant genomes.

### Phylogenetic studies

The phylogenetic tree of the hosted ants using the concatenated nucleotide sequences of three single-copy nuclear genes (Fig. 2) is consistent with the ant phylogenies reported by several authors^31,32^. However, the phylogenetic relationships among species of the same genus are not well-known and even for some genera, their taxonomy has been discussed^32^. For example, it is accepted that *Messor* and *Aphaenogaster* are closely related genera, but both are probably polyphyletic. This would explain why species of both genera do not separate into two different groups. In any case, the species from the same subfamily cluster together in the same group (Fig. 2). When a single species of a subfamily has been studied, nuclear markers cluster it with species belonging to the closest subfamily. For instance, *Myrmecia croslandi* and *Pseudomyrmex gracilis* appear together in the phylogenetic tree. These ants belong to the subfamilies Myrmicinae and Pseudomyrmicinae, which are close together, according to different authors^31,32^.

On the contrary, in the phylogenetic tree drawn for *mariner* elements, the elements hosted by each ant species are not clustered together. Therefore, this tree is highly inconsistent with the phylogeny of the host ants obtained using nuclear genes, with almost identical *mariner* elements found in clearly distantly related species and, on the contrary, more variable *mariner* elements found in closely related species. For example, the *mariners* isolated in the primitive *Leptanilla ortunoi* (Leptanillinae) are included in Clade I with the *mariners* isolated in *Aphaenogaster cardenai* (Myrmicinae). However, these species belong to evolutionarily very distant subfamilies. The opposite happens, for instance, in the isolated *mariners* from the three *Aphaenogaster* species. Together, the inconsistency between the phylogeny obtained with nuclear markers and the phylogenetic analysis using *mariner* sequences indicates that *mariners* have evolved independently of speciation events of the ants that host them. Similar results have been found by others authors for *Drosophila* species^20^.

### Study of genetic relationships among elements and the ants that host them

The majority of the data found in this study suggests the occurrence of horizontal transmission (HT). Rates of neutral evolution in coding genes and TE can be estimated as the average number of nucleotide substitutions at synonymous sites (*K*_S_), assuming that they accumulate at a steady rate. Under vertical transmission, *K*_S_ for a TE and for host species genes will be similar, since both types of sequences have diverged during the same time. Conversely, if the TEs show lower *K*_S_ than the host species genes, divergence times may be different and not comparable, which is a signal of HTs, as is the case in our study. These data clearly suggest the existence of HT events. In some cases, we even found the absence of substitutions at synonymous sites, for example, between *Caubmar-Mb* and *Gconmar-Mb* elements (both included in Subclade II-1) isolated in *Crematogaster auberti* (Myrmicinae) and *Gnamptogenys continua* (Ectatomminae). The clades that gave rise to both subfamilies separated about 90–125 Mya ago^30,31^. The absence of substitutions at synonymous sites between TEs from different species has also been found in other TEs^33^ and has been related to horizontal transfer events.

*Mariners* isolated in each *Aphaenogaster* species are included in different groups. The most plausible hypothesis to explain this result raises the possibility of genetic differentiation of the elements before the split of the current host species, and/or the invasion of these species after their separation, as has been suggested by other authors^26^.

### Detection of potentially coding copies

Many of the *mariner-*like sequences analyzed show frame shifts, nonsense mutations, or/and nucleotide deletions, and they are probably non-functional. However, we found potentially coding copies that have an uninterrupted open reading frame (ORF) in eleven species. All have an extremely high sequence identity with *Mboumar* sequences isolated in *M. bouvieri*^26^. Similarly, the putative transposases codified by them show high sequence identities with the active Mboumar-9 transposase^24^. Nevertheless, some copies could be inactive due to mutations in the conserved motif D,D(34)D, which is considered essential for transposase activity, or due to mutations in the 5′ untranslated region, as we noted in the Results section. The potentially coding copies have been isolated in species belonging to the subfamilies Ponerinae, Dorylinae, Dolichoderinae, Formicinae, and Myrmicinae. The clade that includes ponerine ants was separated about 120 Mya ago from the clade that includes the remaining subfamilies^31^. Despite this, the *K*_S_ between *Mboumar* (Myrmicinae) and *Aghimar-Mb* (Ponerinae) is 0.01, which is much lower than the *K*_S_ between *Messor bouvieri* and *Anochetus ghilianii* nuclear genes (0.58). In fact, one of the isolated copies from *Anochetus ghilianii (Aghimar-Mb-*20) shows a very high nucleotide identity with active *Mboumar-9* and codifies a putative transposase with 100% identity with the Mboumar-9 transposase. All these data support the existence of HT events. Some processes, such as purifying selection or convergent evolution, could lead to sequence similarity between different taxa. However, none of these processes could avoid the accumulation of neutral changes after an independent evolution in their respective hosts for millions of years.

### Detection of selection signals in Mboumar-like transposase sequences

The effects and types of selection that act on TEs have been widely discussed and are controversial^34^. Generally, it is accepted that purifying selection acts on TEs^11,20,33,35^, although positive selection may act on transposons in some cases^34^. Szitenberg *et al*.^36^ analyzed the effects of purifying selection in transposons and other factors, such as life history, the mating system, the G + C content, and RNAi pathways, in an effort to try to explain the discrepancy of TE loads in different genomes. They suggested that genetic drift is the main factor in the evolution of TEs. Other authors think that the absence of purifying selection, together with a high identity of sequences and a discontinuous distribution of the element, could be indicative of the horizontal transfer of TEs^28^.

Our study, considering all the obtained data, suggests purifying selection. As indicated in the Introduction, horizontal transmission has been suggested to be under selection since an active element with full enzymatic activity is required. In addition, our results also support purifying selection in sequence groups without active copies, such as Clade II-1. Probably, *Mboumar-lik*e elements invaded ant genomes very recently and have not had time to accumulate mutations, which would explain the obtained results. Purifying selection acting on active and non-active copies has also been reported by other authors^37^.

### Evolutionary history of *Mboumar*-like elements

The evolutionary history of *Mboumar-*like element seems to be very complex. Our results support the existence of many HT events, which have been previously reported in ants^25,26^. We also suggest that cross-mobilization events and/or the amplification of inactive copies by an active transposase encoded by another copy may have been important in this evolutionary process. Mos1 and Mboumar-9 are enzymes with a 68% amino acid identity. However, their ITRs only exhibit a 50% identity^23^. Trubitsyna *et al*.^38^ have shown a minimal cross-recognition of ITR sequences between Mos1 and Mboumar*-*9 transposases given that they retain the specificity of recognition of their own ITRs. All the TEs included in this study conserve the *Mboumar-9* ITRs, which would facilitate cross-mobilization processes.

In many ant species, only inactive *Mboumar* copies have been isolated. For example, all sequences included in Subclade II-1 shared the same deletion of seven nucleotides, causing a stop codon. Nucleotide deletions have been considered a common mechanism of the vertical inactivation of TEs^39^. In addition to the above, this group is characterized by low variability and we found the absence of substitutions at synonymous sites in some cases, which has been related to HT events, as aforementioned. Kharrat *et al*.^39^ reported similar results to those found in Subclade II-1. They studied a *Mos1*-like element isolated in seven species representative of the main tribes of the genus *Aphis* (Hemiptera, Aphidiade). All elements were about 917 bp in length, with a high degree of similarity and shared the same deletion. The authors considered, as a possible hypothesis, the horizontal transfer of inactive copies mobilized by an active transposase codified by another active copy. Nevertheless, the non-detection of active copies led the authors to suggest a vertical transfer of this element, despite its high degree of similarity and the inconsistency between the phylogeny of the host and the TEs. There is a fundamental difference between these results and the ones shown in the present paper. Specifically, we have found 29 potentially active copies hosted in 11 different ant species, although, in some of the groups, no active copies were detected.

Cross-mobilization and amplification of inactive copies could have happened, for instance, in relation to the *Liovmar-Mb*, *Oclamar-Mb*, and *Tnigmar-Mb* inactive elements included in Clade I, as potentially coding copies have been isolated from the ant species that host them (*Lioponera iovis*, *Odontomachus clarus*, and *Tapinoma ibericum*, respectively) (Fig. 1, Table 1).

The most plausible explanation is that the conserved mutations observed in this study were present in very ancient ancestors and have been spread into several ant genomes. Vertical transmission with differential evolutionary success for each of the different groups could also have happened during species-separation processes. During species separation and more recently, frequent HT events could also have occurred. Lineage-sorting or random lineage-sorting processes could also have taken place. In this scenario, the divergence between elements is expected to be lower than that between their hosts. Additionally, closer genetic relationships are expected among elements than among the ants that host them. In addition, in our study, there are data that support the existence of an ancestral polymorphism, such as the existence of elements belonging to different groups in the same host genome. This fact has been explained as a consequence of old element-diversification events in ancient ancestors^11^.

It is also possible that the evolutionary dynamics of *Mboumar*-like elements can be influenced by the genetic system of their ant hosts. The genetic system of host species is considered a significant factor in the dynamics of MLEs^40^. Several authors^41,42^ suggested that the haplodiploid genetic system and the reproductive division of labor (with queens and workers) of the ants reduce the effective size of populations. Population size is also considered an important factor in the evolution of TEs. In small populations, genetic drift may allow the accumulation of slightly deleterious mutations. As a result, the transposons would not be removed from the genome and would become fixed in the host population^43^. Groth and Blumenstiel^44^ have reported that “In small populations, mildly deleterious TE insertion alleles are allowed to fix, leading to increased copy number”. In a similar way, Wallau *et al*.^9^ suggested that in several tetrapod species with small population sizes, genetic drift would increase the probability of a new transposon being fixed in the host genome.

Until recently, horizontal transmission has been considered a rare event. However, in recent years, various studies have shown that HT is more common than previously thought^45^. At the same time, the importance of TEs in the evolution of eukaryotic genomes has been recognized^46,47^. Specifically, in the ant *Cardiocondyla obscuratior*, the study of its genome suggests an important role of TEs in the adaptation processes that have occurred in this ant^48^. Additionally, Peccoud *et al*.^49^ have suggested that up to 24% of all nucleotides in the insect genome could have been generated by HT events, which is indicative of the relevant role of the HT of TEs in insect genome evolution.

We suggest a complex evolutionary history of this element with vertical transfer and a large horizontal transfer component. In this regard, in support of the above, we recall that only three active transposons have been found to date: *Mos1*^50^, *Famar1* from *Forficula auricularia*^51^, and *Mboumar-9* from *Messor bouvieri*^24^. In spite of all the above, new studies are necessary to fully clarify the complex evolutionary process of *Mboumar-like* elements.

## Conclusions

*Mbouma*r-9 is an active *mariner* TE previously isolated in the ant *Messor bouvieri*. *Mboumar*-like elements isolated from 22 species of ants, belonging to nine different ant subfamilies, have been analyzed. Consequently, *Mboumar*-like elements seem to be well-represented in ant genomes. The phylogenetic tree drawn for *mariner* elements is highly inconsistent with the phylogeny of the host ants. In addition, we found closer genetic relationships among the elements than among the ants that host them. Potential coding copies with an uninterrupted open reading frame of 345 aa have been found in 11 species. Furthermore, a discontinuous presence of *Mboumar-*like elements is found in the ant genomes. Our study suggests a complex evolutionary history of the *Mboumar*-like *mariner* in ants, with important participation of horizontal transfer events. We also suggest that the evolutionary dynamics of *Mboumar*-like elements can be influenced by the genetic system of their host ants, which are eusocial insects with a haplodiploid genetic system.

## Material and Methods

### Material, DNA extraction, PCR amplification, and cloning

*Mboumar* elements isolated from *Messor bouvieri* were found for the first time inserted in a miniature inverted-repeat transposable element (MITE)^23^. We designed a single primer using the terminal repeats (ITRs) of this MITE (MEBOTRA: 5′-AGTCAGAAATGACACCTCGATC). A second primer derived from the ITRs of *Mboumar mariner* elements (ITR-MAR: 5′-CCAGGTGTGTCGGTAATTCCTTTCCGG) was also designed to verify the presence of these TEs in ant genomes^25^. The primer MEBOTRA allows the amplification of the MITE and complete sequence of the TEs inserted, including their ITRs. Both primers were used to amplify the *mariners* in *Formica cunicularia*, *Bothriomyrmex saundersi*, and *Anochetus ghillianii*. The sequences obtained using MEBOTRA or ITR-MAR primers are similar and belong to the same subgroup (Table 1 and Fig. S1). However, MEBOTRA was not useful in some species and only amplification with ITR-MAR was obtained. Consequently, we decided to use only this last primer in the other species. All *mariners* amplified with MEBOTRA show that the ITRs are highly conserved, most of them with the same sequence of ITR-MAR primer or only one nucleotide change (exceptionally two changes). Therefore, these sequences could also have been amplified with this second primer. In Fig. S1, the sequences of the ITRs have been deleted when ITR-MAR was used for *mariner* amplification. Although the sequence for the ITRs is real for the amplified *mariners* when the MEBOTRA primer is used, all ITRs have been removed for subsequent analysis.

The presence of *Mboumar mariners* was tested in 48 ant species (Fig. S1). The ant species with positive results are shown in Table 1. Worker ants were sampled in the localities indicated in Table S1. Material previously analyzed as *Tapinoma nigerrimum* is currently considered as *T. ibericum*^52^. Consequently, throughout this work, the name *T. ibericum* was used. However, when we refer to *mariner* sequences of this species, we use *Tnigmar*-Mb, in agreement with the names given in GenBank sequence database.

Pools of five ants from each nest were used to extract genomic DNA using commercial kits (Machery-Nagel GmbH & Co. Düren, Germany). PCR reactions were set up in a 50-µL mixture containing 100 ng of genomic DNA, 0.5 mM of dNTPs, 50 pmol of the primer, and 1 U of Expand High Fidelity Taq polymerase (Roche). PCR mixes were initially denatured at 92 °C for 2 min, using the following cycling profile: 30 cycles at 92 °C (30 s), 50 °C (30 s), and 72 °C (2 min), with a final elongation step of 72 °C for 5 min. PCR reactions without DNA templates were used as a negative control in all PCR amplifications. The amplified fragments were analyzed by electrophoresis in agarose gels, eluted, and cloned into the pGEM-T Easy vector (Promega). Recombinant plasmids were sequenced on both strands by the dideoxy sequencing method. The *mariner* elements found in each species were named following the nomenclature proposed by Robertson and Asplund^53^, with the modification of Lorite *et al*.^25^.

The phylogeny of the host ants was approximated by using the concatenated nucleotide sequences from three nuclear gene fragments: *wingless* (*wnt-1*), *abdominal-A* (*abdA*), and *long-wavelength rhodopsin* (*lw-Rh*). For species with available data, sequences were retrieved from GenBank, whereas for species whose data were not available, these three genes were amplified following the methodology described by Lorite *et al*.^25^. PCR products were cloned and sequenced as indicated above. In *Gnamptogenys continua* and *Monomorium subopacum*, some sequences were unable to be amplified after repeated attempts. For the analyses, these sequences were replaced by a consensus sequence found for other species of the same genus that were available in GenBank.

### Sequence analyses and molecular evolutionary analyses

Multiple-sequence alignments were performed using CLUSTALW^54^ and BioEdit^55^ and were subsequently corrected by hand in order to maintain the open reading frame (ORF). Sequence comparisons, ORF searches, and other sequence analyses were performed using available online programs from NCBI (http://www.ncbi.nlm.nih.gov/guide/).

The nucleotide substitution models were evaluated using MEGA version X^56^. The models with the lowest Bayesian Information Criterion (BIC) scores were considered the best for describing the substitution pattern. Nucleotide diversities between *Mboumar*-like elements in the different ant hosts were estimated using the T92 + G model run with the MEGA X program.

The phylogenetic relationships among the *mariner* sequences were analyzed using maximum-likelihood (ML) methods. We used the consensus sequences of *Myrmar* and *Tnigmar-Mr mariner* elements isolated from the ants *Myrmica ruginodis*^21^ and *Tapinoma ibericum*^25^ as out-groups, both of which belong to the *mauritiana* subfamily. We also used *Mos1* as the out-group, which is the best-known element of the *mauritiana* subfamily. An ML tree was constructed using a T92 + G run with the MEGA X program. Bootstrap values for each branch were assessed from 1000 replicates in both cases.

The phylogenetic relationships among the host ants were analyzed using the concatenated nucleotide sequences from three nuclear genes fragments, as indicated above. The nucleotide substitution model used was T92 + G + I. The model also runs with the MEGA program X. We used the sequences from the same nuclear gene fragments of *Apis mellifera* and *Dasymutilla aureola* as out-groups^57^.

Neutral genetic differentiation was quantified in terms of the average number of nucleotide substitutions at synonymous sites, *K*_S_, which was computed using the modified Nei–Gojobori model using DnaSP v6^58^. Jukes–Cantor (JC) correction^59^ was applied in all cases. The study of the selection and application of the Z-test of selection using the Nei–Gojobori method (Jukes-Cantor) model was performed using MEGA version X and DnaSP v6.

## Supplementary information

Supplementary information.
