# Supplementary material for: Complex Evolutionary History of Mboumar, a Mariner Element Widely Represented in Ant Genomes
Source: Sci Rep. 2020 Feb 13;10:2610. doi: 10.1038/s41598-020-59422-4 (PMC7018970; doi:10.1038/s41598-020-59422-4)
Supplement: Supplementary file 1 — Supplementary information. [file 41598_2020_59422_MOESM1_ESM.pdf]

Supplementary Information

Complex evolutionary history of *Mboumar*, a *mariner* element widely represented in ant genomes

Olivia Sanllorente, Jesús Vela, Pablo Mora, Areli Ruiz-Mena, María Isabel Torres, Pedro Lorite, Teresa Palomeque

Figure S1. Sequence alignment of all *Mboumar*-like elements in relation to the consensus sequence. The numbers refer to the consensus sequence. The inverted terminal repeats (ITR-MAR) are underlined. The ITRs have been deleted in the sequences amplified using ITR-MAR (For more details, see Material and Methods section). The putative TATA box and polyadenylation signal are marked in yellow and green boxes, respectively. The sequences of each group are represented in a different colour (Clade I in blue, Clade II (Subclade II-1) in green and Clade II (remaining sequences) in pink. The non-clustered sequences are shown in purple.

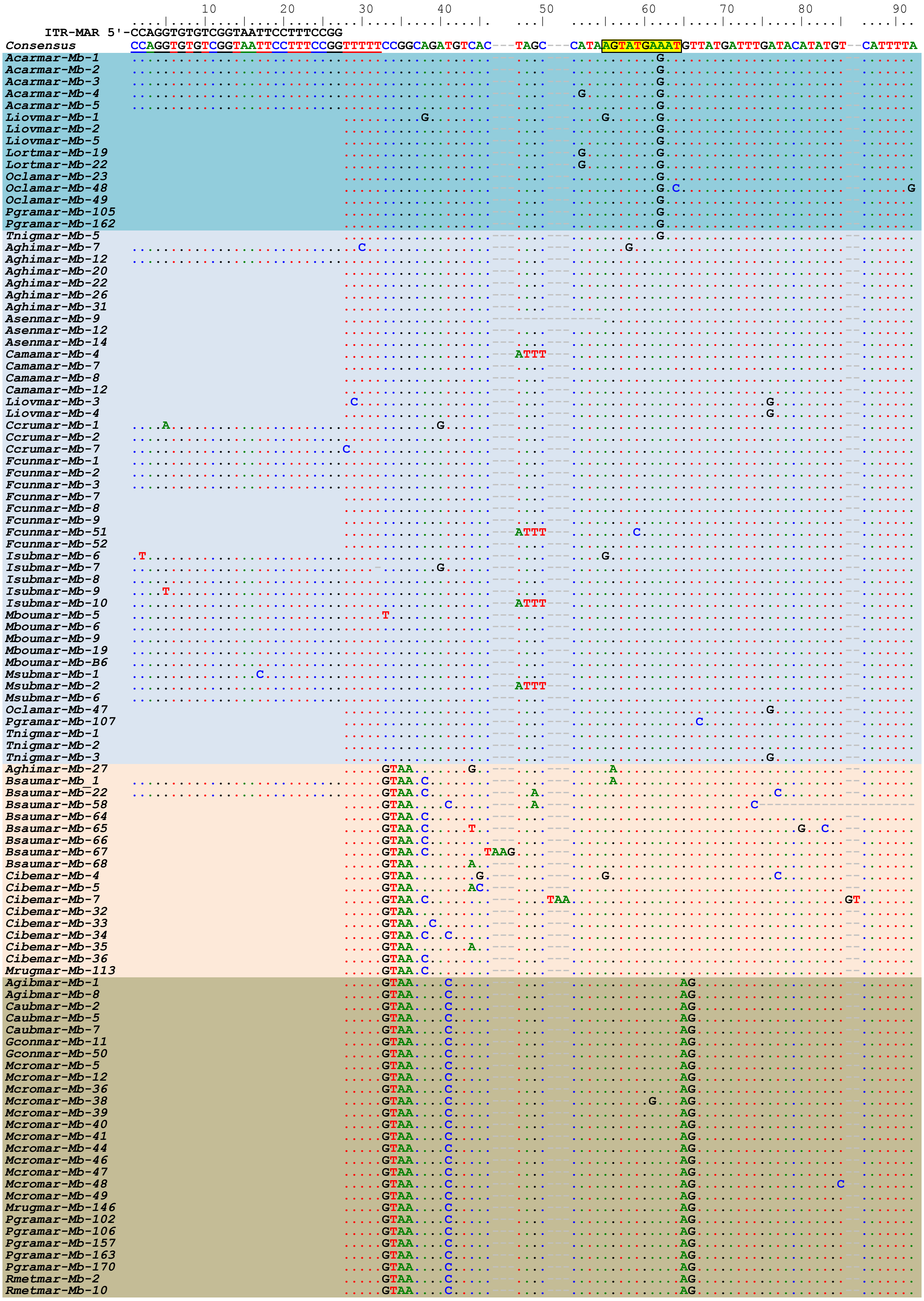

10 20 30 40 50 60 70 80 90
ITR-MAR 5'-CCAGGTGTGTCGGTAATTCCTTTCCGG
Consensus CCAGGTGTGTCGGTAATTCCTTTCCGGTTTTTCCGGCAGATGTCAC---TAGC---CATAAGTATGAAATGTTATGATTTGATACATATGT--CATTTTA
Acarmar-Mb-1
Acarmar-Mb-2
Acarmar-Mb-3
Acarmar-Mb-4
Acarmar-Mb-5
Liovmar-Mb-1
Liovmar-Mb-2
Liovmar-Mb-5
Lortmar-Mb-19
Lortmar-Mb-22
Oclamar-Mb-23
Oclamar-Mb-48
Oclamar-Mb-49
Pgramar-Mb-105
Pgramar-Mb-162
Tnigmar-Mb-5
Aghimar-Mb-7
Aghimar-Mb-12
Aghimar-Mb-20
Aghimar-Mb-22
Aghimar-Mb-26
Aghimar-Mb-31
Asenmar-Mb-9
Asenmar-Mb-12
Asenmar-Mb-14
Camamar-Mb-4
Camamar-Mb-7
Camamar-Mb-8
Camamar-Mb-12
Liovmar-Mb-3
Liovmar-Mb-4
Ccrumar-Mb-1
Ccrumar-Mb-2
Ccrumar-Mb-7
Fcunmar-Mb-1
Fcunmar-Mb-2
Fcunmar-Mb-3
Fcunmar-Mb-7
Fcunmar-Mb-8
Fcunmar-Mb-9
Fcunmar-Mb-51
Fcunmar-Mb-52
Isubmar-Mb-6
Isubmar-Mb-7
Isubmar-Mb-8
Isubmar-Mb-9
Isubmar-Mb-10
Mboumar-Mb-5
Mboumar-Mb-6
Mboumar-Mb-9
Mboumar-Mb-19
Mboumar-Mb-B6
Msubmar-Mb-1
Msubmar-Mb-2
Msubmar-Mb-6
Oclamar-Mb-47
Pgramar-Mb-107
Tnigmar-Mb-1
Tnigmar-Mb-2
Tnigmar-Mb-3
Aghimar-Mb-27
Bsaumar-Mb_1
Bsaumar-Mb-22
Bsaumar-Mb-58
Bsaumar-Mb-64
Bsaumar-Mb-65
Bsaumar-Mb-66
Bsaumar-Mb-67
Bsaumar-Mb-68
Cibemar-Mb-4
Cibemar-Mb-5
Cibemar-Mb-7
Cibemar-Mb-32
Cibemar-Mb-33
Cibemar-Mb-34
Cibemar-Mb-35
Cibemar-Mb-36
Mrugmar-Mb-113
Agibmar-Mb-1
Agibmar-Mb-8
Caubmar-Mb-2
Caubmar-Mb-5
Caubmar-Mb-7
Gconmar-Mb-11
Gconmar-Mb-50
Mcromar-Mb-5
Mcromar-Mb-12
Mcromar-Mb-36
Mcromar-Mb-38
Mcromar-Mb-39
Mcromar-Mb-40
Mcromar-Mb-41
Mcromar-Mb-44
Mcromar-Mb-46
Mcromar-Mb-47
Mcromar-Mb-48
Mcromar-Mb-49
Mrugmar-Mb-146
Pgramar-Mb-102
Pgramar-Mb-106
Pgramar-Mb-157
Pgramar-Mb-163
Pgramar-Mb-170
Rmetmar-Mb-2
Rmetmar-Mb-10

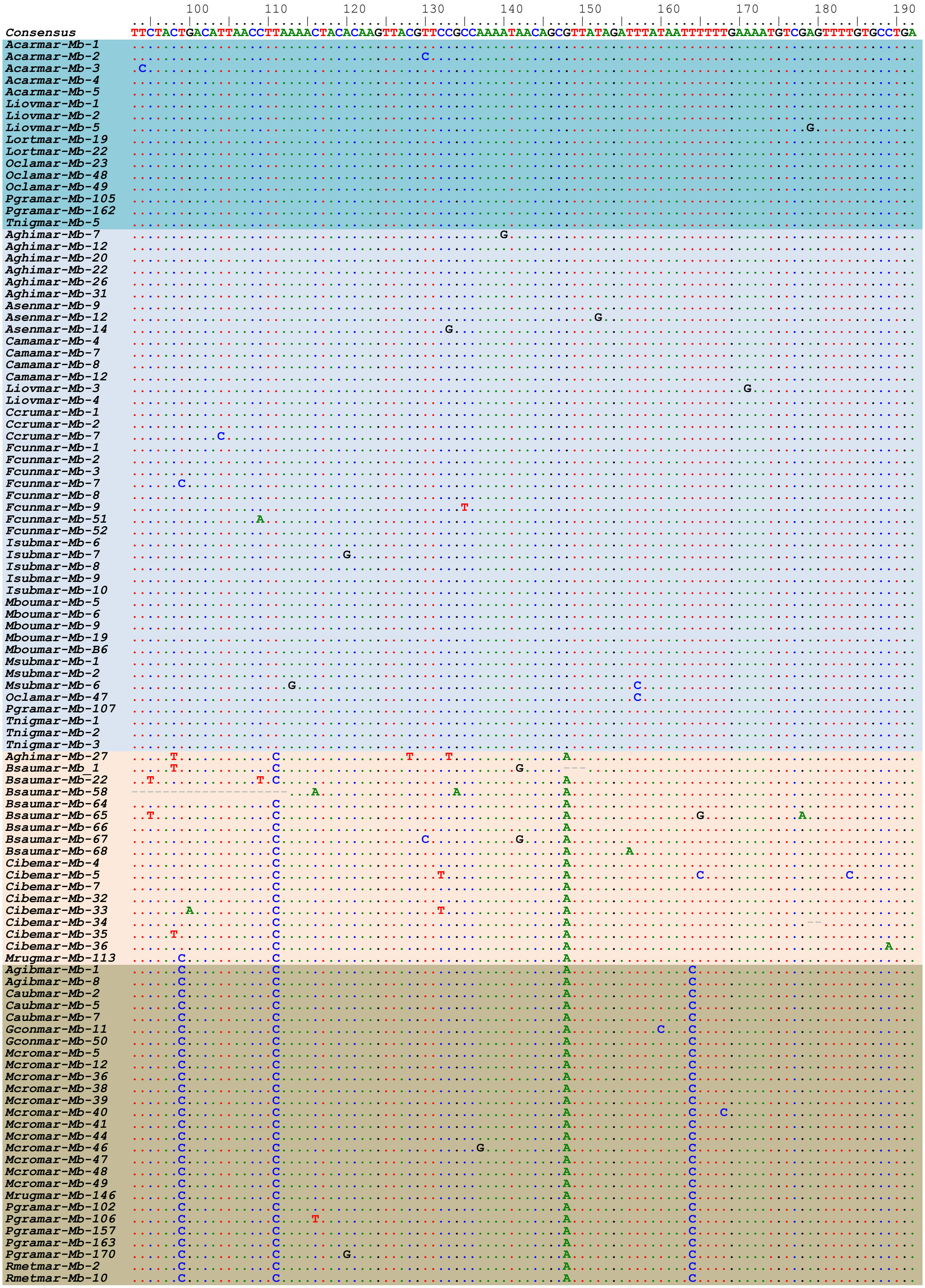
100 110 120 130 140 150 160 170 180 190
Consensus
TTCTACTGACATTAACCTTAAAACTACACAAGTTACGTTCCGCCAAAATAACAGCGTTATAGATTTATAATTTTTTGAAAATGTCGAGTTTTGTGCCTGA
Acarmar-Mb-1
Acarmar-Mb-2
Acarmar-Mb-3
Acarmar-Mb-4
Acarmar-Mb-5
Liovmar-Mb-1
Liovmar-Mb-2
Liovmar-Mb-5
Lortmar-Mb-19
Lortmar-Mb-22
Oclamar-Mb-23
Oclamar-Mb-48
Oclamar-Mb-49
Pgramar-Mb-105
Pgramar-Mb-162
Tnigmar-Mb-5
Aghimar-Mb-7
Aghimar-Mb-12
Aghimar-Mb-20
Aghimar-Mb-22
Aghimar-Mb-26
Aghimar-Mb-31
Asenmar-Mb-9
Asenmar-Mb-12
Asenmar-Mb-14
Camamar-Mb-4
Camamar-Mb-7
Camamar-Mb-8
Camamar-Mb-12
Liovmar-Mb-3
Liovmar-Mb-4
Ccrumar-Mb-1
Ccrumar-Mb-2
Ccrumar-Mb-7
Fcunmar-Mb-1
Fcunmar-Mb-2
Fcunmar-Mb-3
Fcunmar-Mb-7
Fcunmar-Mb-8
Fcunmar-Mb-9
Fcunmar-Mb-51
Fcunmar-Mb-52
Isubmar-Mb-6
Isubmar-Mb-7
Isubmar-Mb-8
Isubmar-Mb-9
Isubmar-Mb-10
Mboumar-Mb-5
Mboumar-Mb-6
Mboumar-Mb-9
Mboumar-Mb-19
Mboumar-Mb-B6
Msubmar-Mb-1
Msubmar-Mb-2
Msubmar-Mb-6
Oclamar-Mb-47
Pgramar-Mb-107
Tnigmar-Mb-1
Tnigmar-Mb-2
Tnigmar-Mb-3
Aghimar-Mb-27
Bsaumar-Mb_1
Bsaumar-Mb-22
Bsaumar-Mb-58
Bsaumar-Mb-64
Bsaumar-Mb-65
Bsaumar-Mb-66
Bsaumar-Mb-67
Bsaumar-Mb-68
Cibemar-Mb-4
Cibemar-Mb-5
Cibemar-Mb-7
Cibemar-Mb-32
Cibemar-Mb-33
Cibemar-Mb-34
Cibemar-Mb-35
Cibemar-Mb-36
Mrugmar-Mb-113
Agibmar-Mb-1
Agibmar-Mb-8
Caubmar-Mb-2
Caubmar-Mb-5
Caubmar-Mb-7
Gconmar-Mb-11
Gconmar-Mb-50
Mcromar-Mb-5
Mcromar-Mb-12
Mcromar-Mb-36
Mcromar-Mb-38
Mcromar-Mb-39
Mcromar-Mb-40
Mcromar-Mb-41
Mcromar-Mb-44
Mcromar-Mb-46
Mcromar-Mb-47
Mcromar-Mb-48
Mcromar-Mb-49
Mrugmar-Mb-146
Pgramar-Mb-102
Pgramar-Mb-106
Pgramar-Mb-157
Pgramar-Mb-163
Pgramar-Mb-170
Rmetmar-Mb-2
Rmetmar-Mb-10

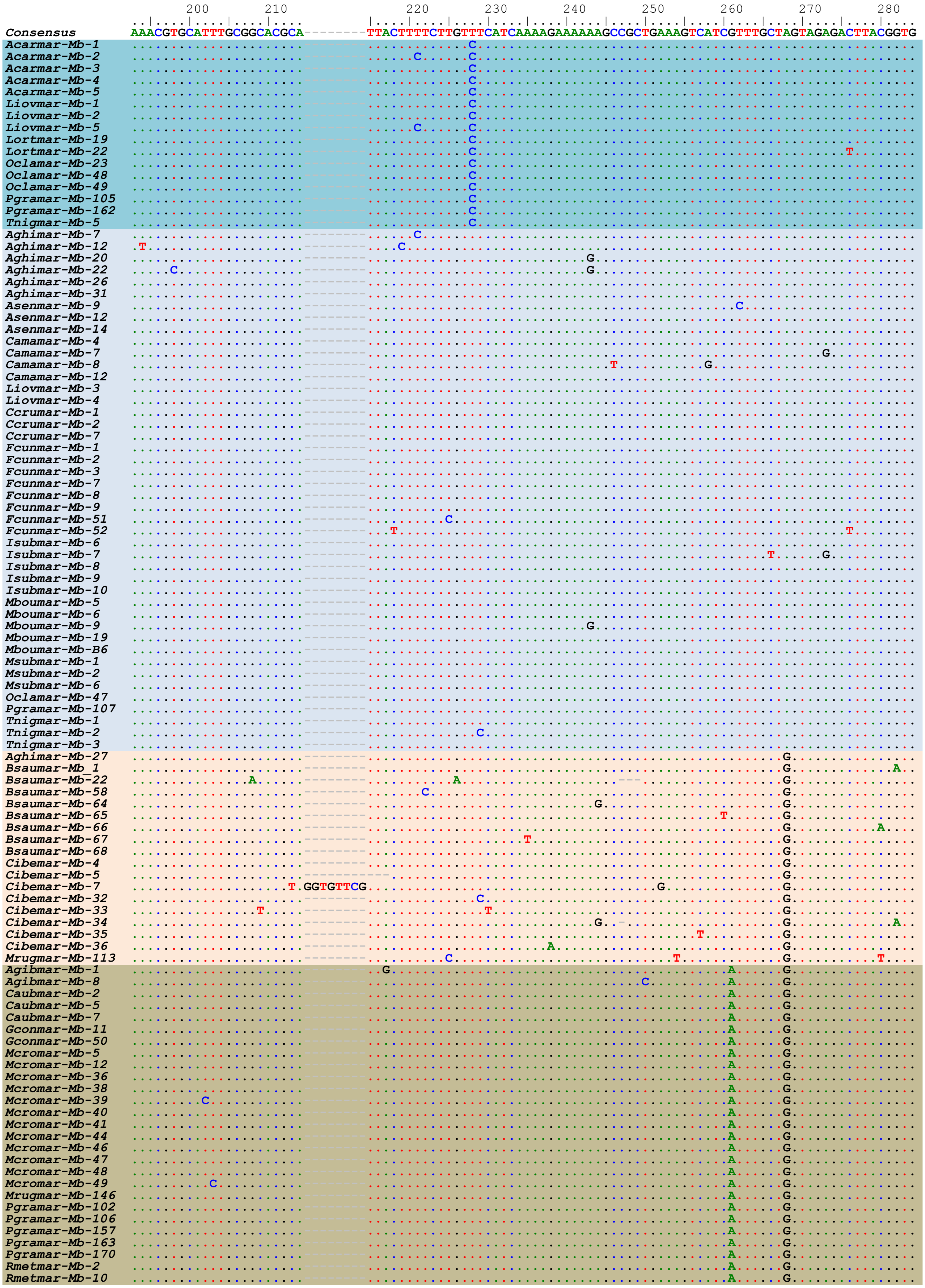

200
210
220
230
240
250
260
270
280
Consensus
AAACGTGCATTTGCGGCACGCA-------TTACTTTTCTTGTTTCATCAAAAGAAAAAAGCCGCTGAAAGTCATCGTTTGCTAGTAGAGACTTACGGTG
Acarmar-Mb-1
Acarmar-Mb-2
Acarmar-Mb-3
Acarmar-Mb-4
Acarmar-Mb-5
Liovmar-Mb-1
Liovmar-Mb-2
Liovmar-Mb-5
Lortmar-Mb-19
Lortmar-Mb-22
Oclamar-Mb-23
Oclamar-Mb-48
Oclamar-Mb-49
Pgramar-Mb-105
Pgramar-Mb-162
Tnigmar-Mb-5
Aghimar-Mb-7
Aghimar-Mb-12
Aghimar-Mb-20
Aghimar-Mb-22
Aghimar-Mb-26
Aghimar-Mb-31
Asenmar-Mb-9
Asenmar-Mb-12
Asenmar-Mb-14
Camamar-Mb-4
Camamar-Mb-7
Camamar-Mb-8
Camamar-Mb-12
Liovmar-Mb-3
Liovmar-Mb-4
Ccrumar-Mb-1
Ccrumar-Mb-2
Ccrumar-Mb-7
Fcunmar-Mb-1
Fcunmar-Mb-2
Fcunmar-Mb-3
Fcunmar-Mb-7
Fcunmar-Mb-8
Fcunmar-Mb-9
Fcunmar-Mb-51
Fcunmar-Mb-52
Isubmar-Mb-6
Isubmar-Mb-7
Isubmar-Mb-8
Isubmar-Mb-9
Isubmar-Mb-10
Mboumar-Mb-5
Mboumar-Mb-6
Mboumar-Mb-9
Mboumar-Mb-19
Mboumar-Mb-B6
Msubmar-Mb-1
Msubmar-Mb-2
Msubmar-Mb-6
Oclamar-Mb-47
Pgramar-Mb-107
Tnigmar-Mb-1
Tnigmar-Mb-2
Tnigmar-Mb-3
Aghimar-Mb-27
Bsaumar-Mb_1
Bsaumar-Mb-22
Bsaumar-Mb-58
Bsaumar-Mb-64
Bsaumar-Mb-65
Bsaumar-Mb-66
Bsaumar-Mb-67
Bsaumar-Mb-68
Cibemar-Mb-4
Cibemar-Mb-5
Cibemar-Mb-7
Cibemar-Mb-32
Cibemar-Mb-33
Cibemar-Mb-34
Cibemar-Mb-35
Cibemar-Mb-36
Mrugmar-Mb-113
Agibmar-Mb-1
Agibmar-Mb-8
Caubmar-Mb-2
Caubmar-Mb-5
Caubmar-Mb-7
Gconmar-Mb-11
Gconmar-Mb-50
Mcromar-Mb-5
Mcromar-Mb-12
Mcromar-Mb-36
Mcromar-Mb-38
Mcromar-Mb-39
Mcromar-Mb-40
Mcromar-Mb-41
Mcromar-Mb-44
Mcromar-Mb-46
Mcromar-Mb-47
Mcromar-Mb-48
Mcromar-Mb-49
Mrugmar-Mb-146
Pgramar-Mb-102
Pgramar-Mb-106
Pgramar-Mb-157
Pgramar-Mb-163
Pgramar-Mb-170
Rmetmar-Mb-2
Rmetmar-Mb-10

```
                      290       300       310       320       330       340       350       360       370       380
                 |....|....|....|....|....|....|....|....|....|....|....|....|....|....|....|....|....|....|....|....
Consensus        AGCATGCTCCAACCATAAGAACATGTGAAACGTGGTTTCGACAATTCAAATGTGGTGATTTCAACGTTCAAGACAAAGAACGTCCTGGTAGGCCGAAAAC
Acarmar-Mb-1     ....................................................................................................
Acarmar-Mb-2     ....................................................................................................
Acarmar-Mb-3     ....................................................................................................
Acarmar-Mb-4     ....................................................................................................
Acarmar-Mb-5     ....................................................................................................
Liovmar-Mb-1     ....................................................................................................
Liovmar-Mb-2     ....................................................................................................
Liovmar-Mb-5     ....................................................................................................
Lortmar-Mb-19    ....................G.....................................................................G..........
Lortmar-Mb-22    ....................G..............A................A...............................................
Oclamar-Mb-23    ....................................................................................................
Oclamar-Mb-48    ....................................................................................................
Oclamar-Mb-49    ....................................................................................................
Pgramar-Mb-105   ....................................................................................................
Pgramar-Mb-162   ....................................................................................................
Tnigmar-Mb-5     ....................................................................................................
Aghimar-Mb-7     ....................................................................................................
Aghimar-Mb-12    ....................................................................................................
Aghimar-Mb-20    ....................................................................................................
Aghimar-Mb-22    ....................................................................................................
Aghimar-Mb-26    .............................................................................................T......
Aghimar-Mb-31    ....................................................................................................
Asenmar-Mb-9     ....................................................................................................
Asenmar-Mb-12    ....................................................................................................
Asenmar-Mb-14    ....................................................................................................
Camamar-Mb-4     ..................A.................................................................................
Camamar-Mb-7     ....................................................................................................
Camamar-Mb-8     ....................................................................................................
Camamar-Mb-12    ....................................................................................................
Liovmar-Mb-3     ....................................................................................................
Liovmar-Mb-4     .............................G......................................................................
Ccrumar-Mb-1     ....................................................................................................
Ccrumar-Mb-2     ....................................................................................................
Ccrumar-Mb-7     G...................................................................................................
Fcunmar-Mb-1     ....................................................................................................
Fcunmar-Mb-2     ....................................................................................................
Fcunmar-Mb-3     ....................................................................................................
Fcunmar-Mb-7     ....................................................................................................
Fcunmar-Mb-8     ....................................................................................................
Fcunmar-Mb-9     ....................................................................................................
Fcunmar-Mb-51    ..................A.................................................................................
Fcunmar-Mb-52    .............................................................T.....................T................
Isubmar-Mb-6     ....................................................................................................
Isubmar-Mb-7     ....................................................................................................
Isubmar-Mb-8     ....................................................................................................
Isubmar-Mb-9     ....................................................................................................
Isubmar-Mb-10    ....C.............A.....................................................................A.........G.
Mboumar-Mb-5     ....................................................................................................
Mboumar-Mb-6     .............................................................................................T......
Mboumar-Mb-9     ....................................................................................................
Mboumar-Mb-19    .............................................................................................T......
Mboumar-Mb-B6    ....................................................................................................
Msubmar-Mb-1     ..................A.................................................................................
Msubmar-Mb-2     ..................A.................................................................................
Msubmar-Mb-6     .............................................................................................T......
Oclamar-Mb-47    ....................................................................................................
Pgramar-Mb-107   ....................................................................................................
Tnigmar-Mb-1     ....................................................................................................
Tnigmar-Mb-2     ....................................................................................................
Tnigmar-Mb-3     ....................................................................................................
Aghimar-Mb-27    ...............................A.......A...........A..............................G.T...............
Bsaumar-Mb_1     ..................................................................................G.................
Bsaumar-Mb-22    ..................................................................................G....A......A.....
Bsaumar-Mb-58    ..................................................................................G.................
Bsaumar-Mb-64    ..................................................................................G...............-.
Bsaumar-Mb-65    .....................................G................A...........................G.T...............
Bsaumar-Mb-66    .......................................A..........................................G.......A.........
Bsaumar-Mb-67    .............T....................................................................G.................
Bsaumar-Mb-68    ..........G...................T..................................................----...............
Cibemar-Mb-4     .........................................................G........................G...........A.....
Cibemar-Mb-5     .................G................................................................G.................
Cibemar-Mb-7     ..................................................................................G..........T......
Cibemar-Mb-32    ..............................A...................................................G.................
Cibemar-Mb-33    ..............................T.........................A....T.........A..........G.......A.........
Cibemar-Mb-34    .........T..T.............................G..........A.......T......T.............GT...............T
Cibemar-Mb-35    ..................................................................................G....A......A.....
Cibemar-Mb-36    ....G..................................................................--.........G.................
Mrugmar-Mb-113   ..................................................................................G........A..A.....
Agibmar-Mb-1     ....C.....................A..........................A............................G.................
Agibmar-Mb-8     ....C.....................A..........................A............................G.................
Caubmar-Mb-2     ....C......G..............A..........................A............................G.................
Caubmar-Mb-5     ....C.....................A..........................A............................G.................
Caubmar-Mb-7     ....C.....................A..........................A............................G.................
Gconmar-Mb-11    ....C.....................A..........................A............................G.................
Gconmar-Mb-50    ....C.....................A..........................A............................G.................
Mcromar-Mb-5     ....C.....................A..........................A............................G.................
Mcromar-Mb-12    ....C.....................A..........................A............................G.................
Mcromar-Mb-36    ....C.....................A..........................A............................G.................
Mcromar-Mb-38    ....C.....................A..........................A............................G.................
Mcromar-Mb-39    ....C.....................A..........................A............................G.................
Mcromar-Mb-40    ....C.....................A..........................A............................G.................
Mcromar-Mb-41    ....C.....................A..........................A............................G.................
Mcromar-Mb-44    ....C.....................A..........................A............................G.................
Mcromar-Mb-46    ....C.....................A..........................A............................G.................
Mcromar-Mb-47    ....C.....................A..........................A............................G.................
Mcromar-Mb-48    ....C.....................A..........................A............................G.................
Mcromar-Mb-49    ....C.....................A..........................A............................G.................
Mrugmar-Mb-146   ....C.....................A..........................A............................G.................
Pgramar-Mb-102   ....C.....................A..........................A............................G...............G.
Pgramar-Mb-106   ....C.....................A..........................A............................G.................
Pgramar-Mb-157   ....C.....................A..........................A............................G.................
Pgramar-Mb-163   ....C.....................A..........................A............................G.................
Pgramar-Mb-170   ....C.....................A..........................A............................G.................
Rmetmar-Mb-2     ....C.....................A..........................A............................G.................
Rmetmar-Mb-10    ....C.....................A..........................A............................G.................
```

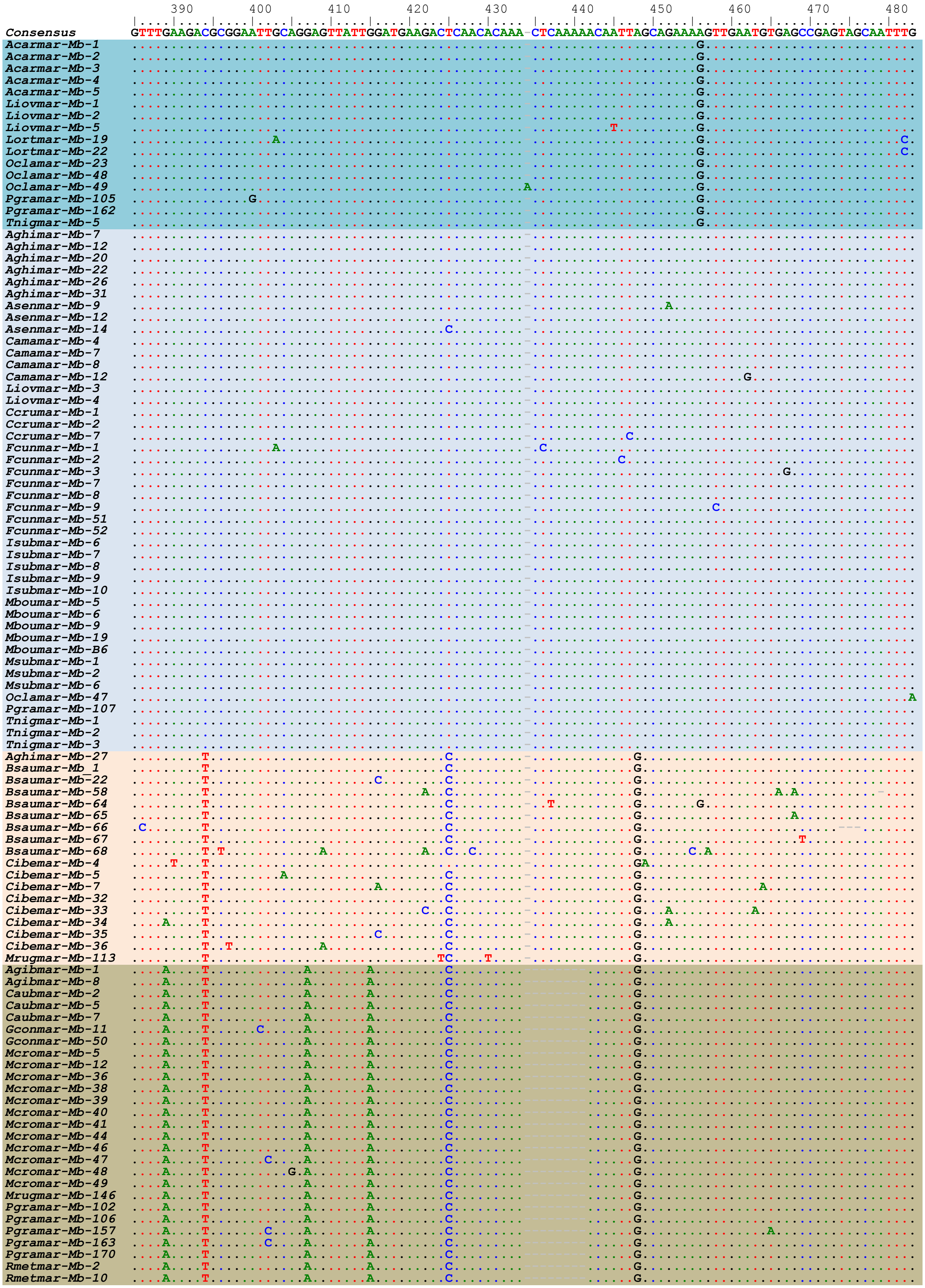
390
400
410
420
430
440
450
460
470
480
Consensus
GTTTGAAGACGCGGAATTGCAGGAGTTATTGGATGAAGACTCAACACAAA-CTCAAAAACAATTAGCAGAAAAGTTGAATGTGAGCCGAGTAGCAATTTG
Acarmar-Mb-1
Acarmar-Mb-2
Acarmar-Mb-3
Acarmar-Mb-4
Acarmar-Mb-5
Liovmar-Mb-1
Liovmar-Mb-2
Liovmar-Mb-5
Lortmar-Mb-19
Lortmar-Mb-22
Oclamar-Mb-23
Oclamar-Mb-48
Oclamar-Mb-49
Pgramar-Mb-105
Pgramar-Mb-162
Tnigmar-Mb-5
Aghimar-Mb-7
Aghimar-Mb-12
Aghimar-Mb-20
Aghimar-Mb-22
Aghimar-Mb-26
Aghimar-Mb-31
Asenmar-Mb-9
Asenmar-Mb-12
Asenmar-Mb-14
Camamar-Mb-4
Camamar-Mb-7
Camamar-Mb-8
Camamar-Mb-12
Liovmar-Mb-3
Liovmar-Mb-4
Ccrumar-Mb-1
Ccrumar-Mb-2
Ccrumar-Mb-7
Fcunmar-Mb-1
Fcunmar-Mb-2
Fcunmar-Mb-3
Fcunmar-Mb-7
Fcunmar-Mb-8
Fcunmar-Mb-9
Fcunmar-Mb-51
Fcunmar-Mb-52
Isubmar-Mb-6
Isubmar-Mb-7
Isubmar-Mb-8
Isubmar-Mb-9
Isubmar-Mb-10
Mboumar-Mb-5
Mboumar-Mb-6
Mboumar-Mb-9
Mboumar-Mb-19
Mboumar-Mb-B6
Msubmar-Mb-1
Msubmar-Mb-2
Msubmar-Mb-6
Oclamar-Mb-47
Pgramar-Mb-107
Tnigmar-Mb-1
Tnigmar-Mb-2
Tnigmar-Mb-3
Aghimar-Mb-27
Bsaumar-Mb_1
Bsaumar-Mb-22
Bsaumar-Mb-58
Bsaumar-Mb-64
Bsaumar-Mb-65
Bsaumar-Mb-66
Bsaumar-Mb-67
Bsaumar-Mb-68
Cibemar-Mb-4
Cibemar-Mb-5
Cibemar-Mb-7
Cibemar-Mb-32
Cibemar-Mb-33
Cibemar-Mb-34
Cibemar-Mb-35
Cibemar-Mb-36
Mrugmar-Mb-113
Agibmar-Mb-1
Agibmar-Mb-8
Caubmar-Mb-2
Caubmar-Mb-5
Caubmar-Mb-7
Gconmar-Mb-11
Gconmar-Mb-50
Mcromar-Mb-5
Mcromar-Mb-12
Mcromar-Mb-36
Mcromar-Mb-38
Mcromar-Mb-39
Mcromar-Mb-40
Mcromar-Mb-41
Mcromar-Mb-44
Mcromar-Mb-46
Mcromar-Mb-47
Mcromar-Mb-48
Mcromar-Mb-49
Mrugmar-Mb-146
Pgramar-Mb-102
Pgramar-Mb-106
Pgramar-Mb-157
Pgramar-Mb-163
Pgramar-Mb-170
Rmetmar-Mb-2
Rmetmar-Mb-10

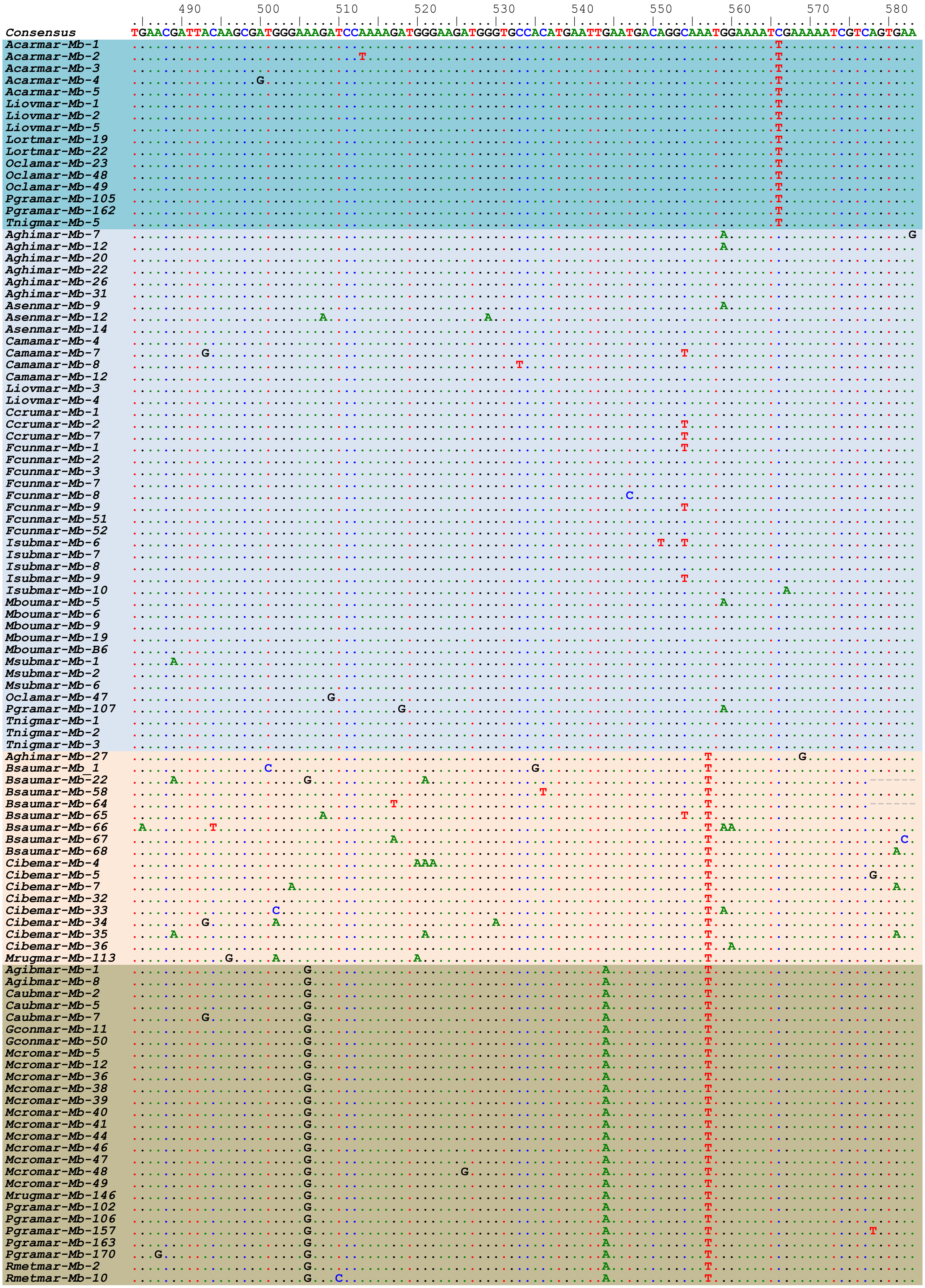
490 500 510 520 530 540 550 560 570 580
Consensus
TGAACGATTACAAGCGATGGGAAAGATCCAAAAGATGGGAAGATGGGTGCCACATGAATTGAATGACAGGCAAATGGAAAATCGAAAAATCGTCAGTGAA
Acarmar-Mb-1
Acarmar-Mb-2
Acarmar-Mb-3
Acarmar-Mb-4
Acarmar-Mb-5
Liovmar-Mb-1
Liovmar-Mb-2
Liovmar-Mb-5
Lortmar-Mb-19
Lortmar-Mb-22
Oclamar-Mb-23
Oclamar-Mb-48
Oclamar-Mb-49
Pgramar-Mb-105
Pgramar-Mb-162
Tnigmar-Mb-5
Aghimar-Mb-7
Aghimar-Mb-12
Aghimar-Mb-20
Aghimar-Mb-22
Aghimar-Mb-26
Aghimar-Mb-31
Asenmar-Mb-9
Asenmar-Mb-12
Asenmar-Mb-14
Camamar-Mb-4
Camamar-Mb-7
Camamar-Mb-8
Camamar-Mb-12
Liovmar-Mb-3
Liovmar-Mb-4
Ccrumar-Mb-1
Ccrumar-Mb-2
Ccrumar-Mb-7
Fcunmar-Mb-1
Fcunmar-Mb-2
Fcunmar-Mb-3
Fcunmar-Mb-7
Fcunmar-Mb-8
Fcunmar-Mb-9
Fcunmar-Mb-51
Fcunmar-Mb-52
Isubmar-Mb-6
Isubmar-Mb-7
Isubmar-Mb-8
Isubmar-Mb-9
Isubmar-Mb-10
Mboumar-Mb-5
Mboumar-Mb-6
Mboumar-Mb-9
Mboumar-Mb-19
Mboumar-Mb-B6
Msubmar-Mb-1
Msubmar-Mb-2
Msubmar-Mb-6
Oclamar-Mb-47
Pgramar-Mb-107
Tnigmar-Mb-1
Tnigmar-Mb-2
Tnigmar-Mb-3
Aghimar-Mb-27
Bsaumar-Mb_1
Bsaumar-Mb-22
Bsaumar-Mb-58
Bsaumar-Mb-64
Bsaumar-Mb-65
Bsaumar-Mb-66
Bsaumar-Mb-67
Bsaumar-Mb-68
Cibemar-Mb-4
Cibemar-Mb-5
Cibemar-Mb-7
Cibemar-Mb-32
Cibemar-Mb-33
Cibemar-Mb-34
Cibemar-Mb-35
Cibemar-Mb-36
Mrugmar-Mb-113
Agibmar-Mb-1
Agibmar-Mb-8
Caubmar-Mb-2
Caubmar-Mb-5
Caubmar-Mb-7
Gconmar-Mb-11
Gconmar-Mb-50
Mcromar-Mb-5
Mcromar-Mb-12
Mcromar-Mb-36
Mcromar-Mb-38
Mcromar-Mb-39
Mcromar-Mb-40
Mcromar-Mb-41
Mcromar-Mb-44
Mcromar-Mb-46
Mcromar-Mb-47
Mcromar-Mb-48
Mcromar-Mb-49
Mrugmar-Mb-146
Pgramar-Mb-102
Pgramar-Mb-106
Pgramar-Mb-157
Pgramar-Mb-163
Pgramar-Mb-170
Rmetmar-Mb-2
Rmetmar-Mb-10

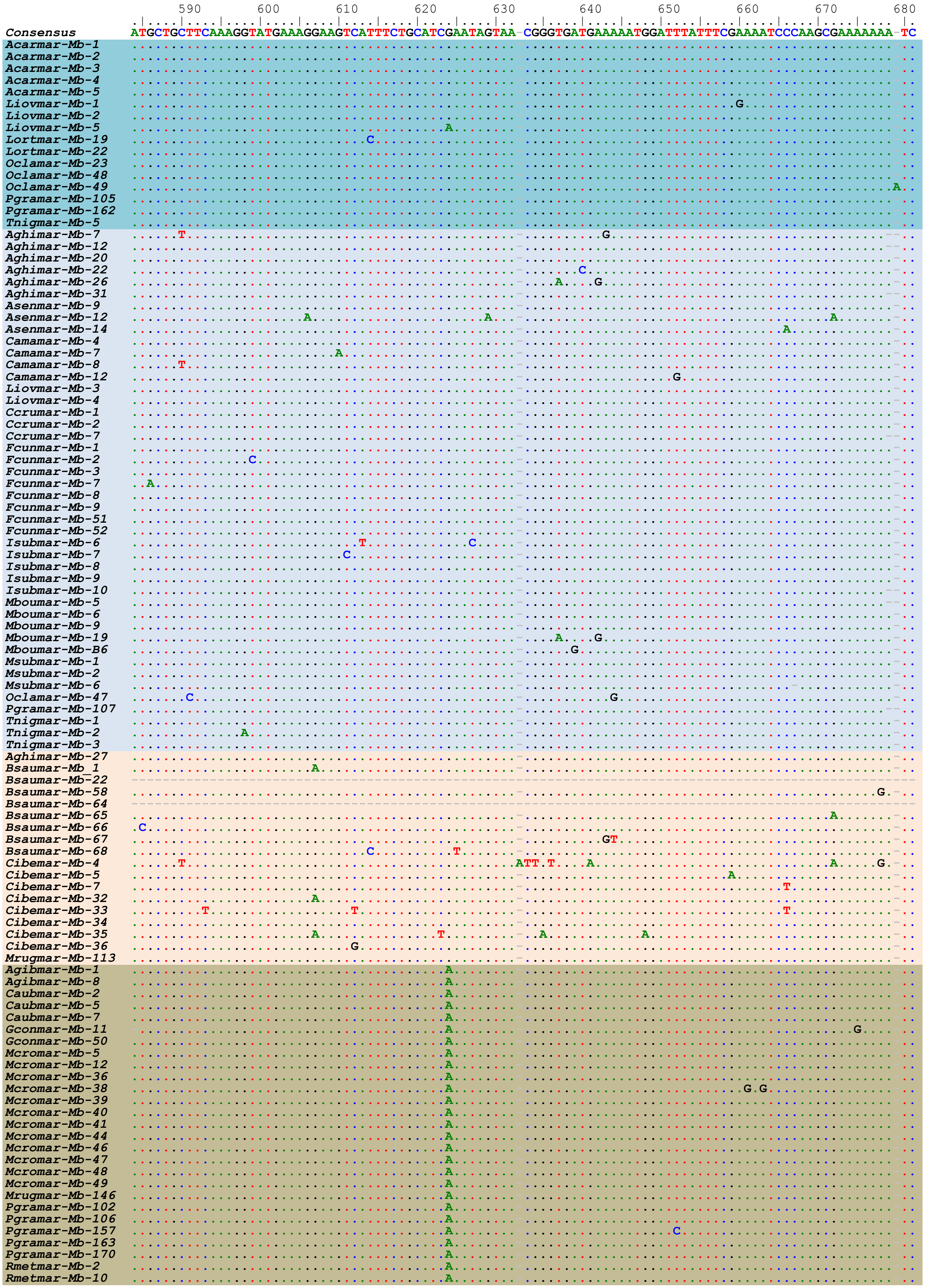
590 600 610 620 630 640 650 660 670 680
Consensus
ATGCTGCTTCAAAGGTATGAAAGGAAGTCATTTCTGCATCGAATAGTAA-CGGGTGATGAAAAATGGATTTATTTCGAAAATCCCAAGCGAAAAAAA-TC
Acarmar-Mb-1
Acarmar-Mb-2
Acarmar-Mb-3
Acarmar-Mb-4
Acarmar-Mb-5
Liovmar-Mb-1
Liovmar-Mb-2
Liovmar-Mb-5
Lortmar-Mb-19
Lortmar-Mb-22
Oclamar-Mb-23
Oclamar-Mb-48
Oclamar-Mb-49
Pgramar-Mb-105
Pgramar-Mb-162
Tnigmar-Mb-5
Aghimar-Mb-7
Aghimar-Mb-12
Aghimar-Mb-20
Aghimar-Mb-22
Aghimar-Mb-26
Aghimar-Mb-31
Asenmar-Mb-9
Asenmar-Mb-12
Asenmar-Mb-14
Camamar-Mb-4
Camamar-Mb-7
Camamar-Mb-8
Camamar-Mb-12
Liovmar-Mb-3
Liovmar-Mb-4
Ccrumar-Mb-1
Ccrumar-Mb-2
Ccrumar-Mb-7
Fcunmar-Mb-1
Fcunmar-Mb-2
Fcunmar-Mb-3
Fcunmar-Mb-7
Fcunmar-Mb-8
Fcunmar-Mb-9
Fcunmar-Mb-51
Fcunmar-Mb-52
Isubmar-Mb-6
Isubmar-Mb-7
Isubmar-Mb-8
Isubmar-Mb-9
Isubmar-Mb-10
Mboumar-Mb-5
Mboumar-Mb-6
Mboumar-Mb-9
Mboumar-Mb-19
Mboumar-Mb-B6
Msubmar-Mb-1
Msubmar-Mb-2
Msubmar-Mb-6
Oclamar-Mb-47
Pgramar-Mb-107
Tnigmar-Mb-1
Tnigmar-Mb-2
Tnigmar-Mb-3
Aghimar-Mb-27
Bsaumar-Mb_1
Bsaumar-Mb-22
Bsaumar-Mb-58
Bsaumar-Mb-64
Bsaumar-Mb-65
Bsaumar-Mb-66
Bsaumar-Mb-67
Bsaumar-Mb-68
Cibemar-Mb-4
Cibemar-Mb-5
Cibemar-Mb-7
Cibemar-Mb-32
Cibemar-Mb-33
Cibemar-Mb-34
Cibemar-Mb-35
Cibemar-Mb-36
Mrugmar-Mb-113
Agibmar-Mb-1
Agibmar-Mb-8
Caubmar-Mb-2
Caubmar-Mb-5
Caubmar-Mb-7
Gconmar-Mb-11
Gconmar-Mb-50
Mcromar-Mb-5
Mcromar-Mb-12
Mcromar-Mb-36
Mcromar-Mb-38
Mcromar-Mb-39
Mcromar-Mb-40
Mcromar-Mb-41
Mcromar-Mb-44
Mcromar-Mb-46
Mcromar-Mb-47
Mcromar-Mb-48
Mcromar-Mb-49
Mrugmar-Mb-146
Pgramar-Mb-102
Pgramar-Mb-106
Pgramar-Mb-157
Pgramar-Mb-163
Pgramar-Mb-170
Rmetmar-Mb-2
Rmetmar-Mb-10

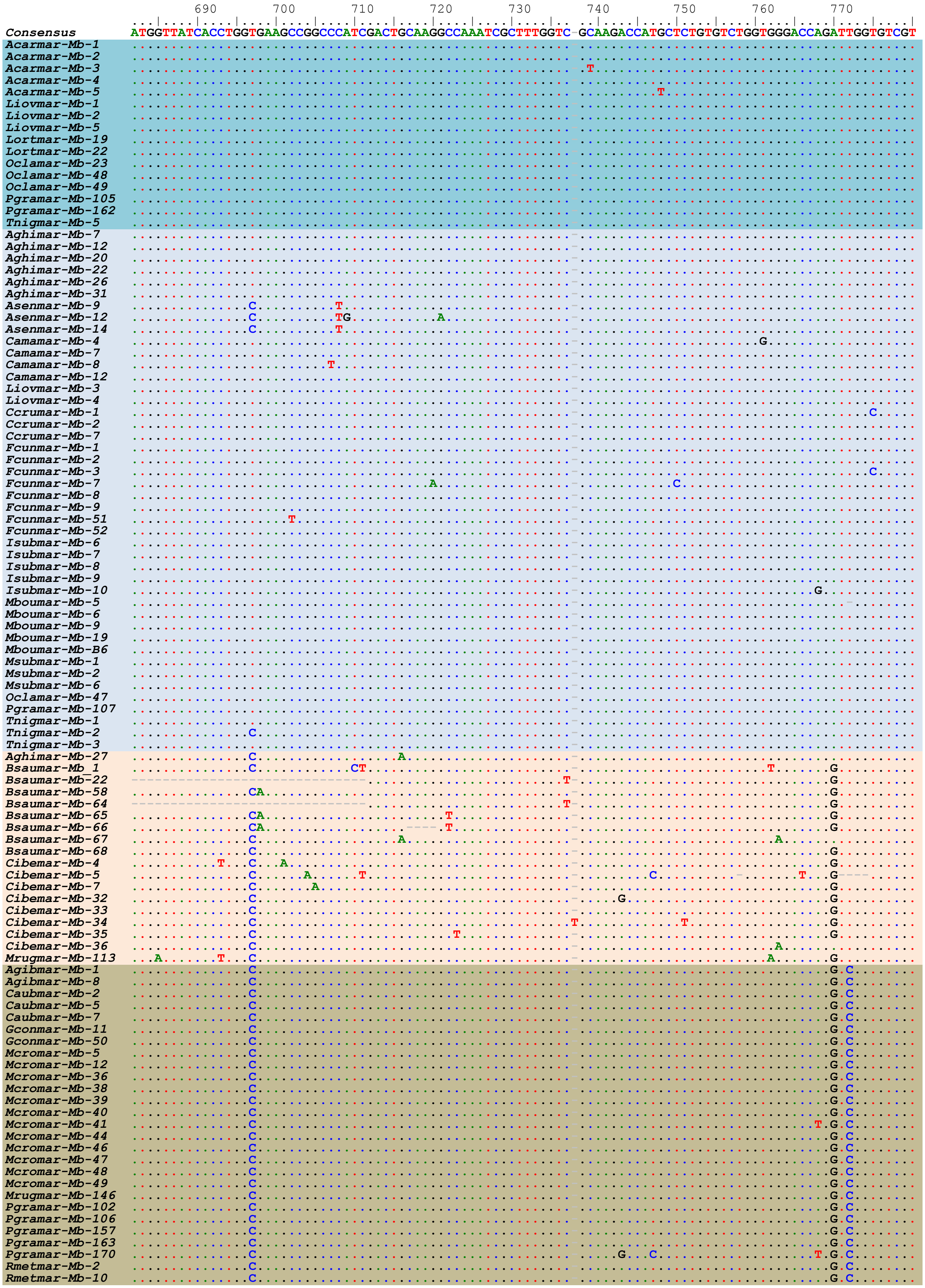

690 700 710 720 730 740 750 760 770
Consensus ATGGTTATCACCTGGTGAAGCCGGCCCATCGACTGCAAGGCCAAATCGCTTTGGTC-GCAAGACCATGCTCTGTGTCTGGTGGGACCAGATTGGTGTCGT
Acarmar-Mb-1
Acarmar-Mb-2
Acarmar-Mb-3
Acarmar-Mb-4
Acarmar-Mb-5
Liovmar-Mb-1
Liovmar-Mb-2
Liovmar-Mb-5
Lortmar-Mb-19
Lortmar-Mb-22
Oclamar-Mb-23
Oclamar-Mb-48
Oclamar-Mb-49
Pgramar-Mb-105
Pgramar-Mb-162
Tnigmar-Mb-5
Aghimar-Mb-7
Aghimar-Mb-12
Aghimar-Mb-20
Aghimar-Mb-22
Aghimar-Mb-26
Aghimar-Mb-31
Asenmar-Mb-9
Asenmar-Mb-12
Asenmar-Mb-14
Camamar-Mb-4
Camamar-Mb-7
Camamar-Mb-8
Camamar-Mb-12
Liovmar-Mb-3
Liovmar-Mb-4
Ccrumar-Mb-1
Ccrumar-Mb-2
Ccrumar-Mb-7
Fcunmar-Mb-1
Fcunmar-Mb-2
Fcunmar-Mb-3
Fcunmar-Mb-7
Fcunmar-Mb-8
Fcunmar-Mb-9
Fcunmar-Mb-51
Fcunmar-Mb-52
Isubmar-Mb-6
Isubmar-Mb-7
Isubmar-Mb-8
Isubmar-Mb-9
Isubmar-Mb-10
Mboumar-Mb-5
Mboumar-Mb-6
Mboumar-Mb-9
Mboumar-Mb-19
Mboumar-Mb-B6
Msubmar-Mb-1
Msubmar-Mb-2
Msubmar-Mb-6
Oclamar-Mb-47
Pgramar-Mb-107
Tnigmar-Mb-1
Tnigmar-Mb-2
Tnigmar-Mb-3
Aghimar-Mb-27
Bsaumar-Mb_1
Bsaumar-Mb-22
Bsaumar-Mb-58
Bsaumar-Mb-64
Bsaumar-Mb-65
Bsaumar-Mb-66
Bsaumar-Mb-67
Bsaumar-Mb-68
Cibemar-Mb-4
Cibemar-Mb-5
Cibemar-Mb-7
Cibemar-Mb-32
Cibemar-Mb-33
Cibemar-Mb-34
Cibemar-Mb-35
Cibemar-Mb-36
Mrugmar-Mb-113
Agibmar-Mb-1
Agibmar-Mb-8
Caubmar-Mb-2
Caubmar-Mb-5
Caubmar-Mb-7
Gconmar-Mb-11
Gconmar-Mb-50
Mcromar-Mb-5
Mcromar-Mb-12
Mcromar-Mb-36
Mcromar-Mb-38
Mcromar-Mb-39
Mcromar-Mb-40
Mcromar-Mb-41
Mcromar-Mb-44
Mcromar-Mb-46
Mcromar-Mb-47
Mcromar-Mb-48
Mcromar-Mb-49
Mrugmar-Mb-146
Pgramar-Mb-102
Pgramar-Mb-106
Pgramar-Mb-157
Pgramar-Mb-163
Pgramar-Mb-170
Rmetmar-Mb-2
Rmetmar-Mb-10

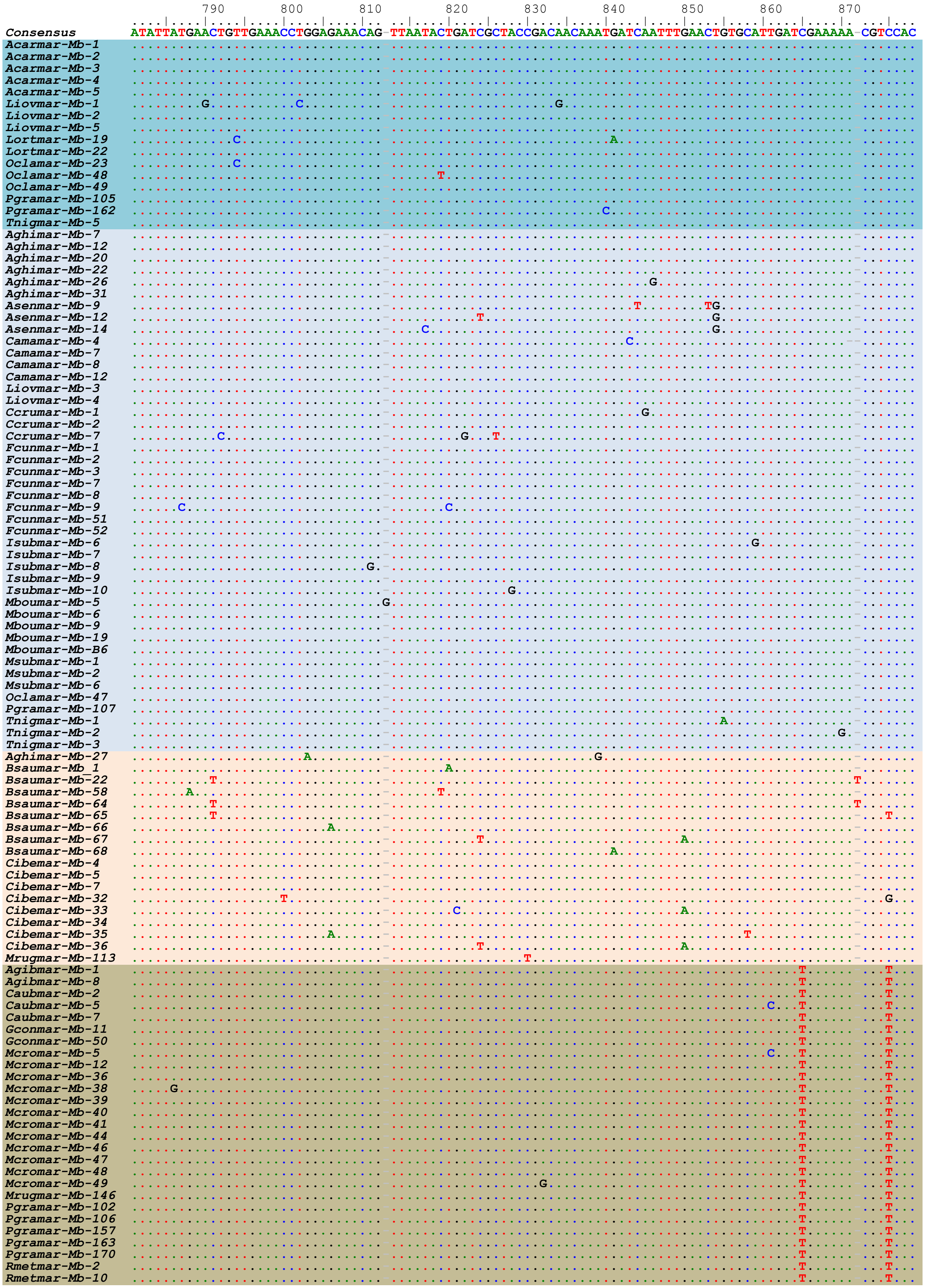

790 800 810 820 830 840 850 860 870
Consensus
ATATTATGAACTGTTGAAACCTGGAGAAACAG-TTAATACTGATCGCTACCGACAACAAATGATCAATTTGAACTGTGCATTGATCGAAAAA-CGTCCAC
Acarmar-Mb-1
Acarmar-Mb-2
Acarmar-Mb-3
Acarmar-Mb-4
Acarmar-Mb-5
Liovmar-Mb-1
Liovmar-Mb-2
Liovmar-Mb-5
Lortmar-Mb-19
Lortmar-Mb-22
Oclamar-Mb-23
Oclamar-Mb-48
Oclamar-Mb-49
Pgramar-Mb-105
Pgramar-Mb-162
Tnigmar-Mb-5
Aghimar-Mb-7
Aghimar-Mb-12
Aghimar-Mb-20
Aghimar-Mb-22
Aghimar-Mb-26
Aghimar-Mb-31
Asenmar-Mb-9
Asenmar-Mb-12
Asenmar-Mb-14
Camamar-Mb-4
Camamar-Mb-7
Camamar-Mb-8
Camamar-Mb-12
Liovmar-Mb-3
Liovmar-Mb-4
Ccrumar-Mb-1
Ccrumar-Mb-2
Ccrumar-Mb-7
Fcunmar-Mb-1
Fcunmar-Mb-2
Fcunmar-Mb-3
Fcunmar-Mb-7
Fcunmar-Mb-8
Fcunmar-Mb-9
Fcunmar-Mb-51
Fcunmar-Mb-52
Isubmar-Mb-6
Isubmar-Mb-7
Isubmar-Mb-8
Isubmar-Mb-9
Isubmar-Mb-10
Mboumar-Mb-5
Mboumar-Mb-6
Mboumar-Mb-9
Mboumar-Mb-19
Mboumar-Mb-B6
Msubmar-Mb-1
Msubmar-Mb-2
Msubmar-Mb-6
Oclamar-Mb-47
Pgramar-Mb-107
Tnigmar-Mb-1
Tnigmar-Mb-2
Tnigmar-Mb-3
Aghimar-Mb-27
Bsaumar-Mb_1
Bsaumar-Mb-22
Bsaumar-Mb-58
Bsaumar-Mb-64
Bsaumar-Mb-65
Bsaumar-Mb-66
Bsaumar-Mb-67
Bsaumar-Mb-68
Cibemar-Mb-4
Cibemar-Mb-5
Cibemar-Mb-7
Cibemar-Mb-32
Cibemar-Mb-33
Cibemar-Mb-34
Cibemar-Mb-35
Cibemar-Mb-36
Mrugmar-Mb-113
Agibmar-Mb-1
Agibmar-Mb-8
Caubmar-Mb-2
Caubmar-Mb-5
Caubmar-Mb-7
Gconmar-Mb-11
Gconmar-Mb-50
Mcromar-Mb-5
Mcromar-Mb-12
Mcromar-Mb-36
Mcromar-Mb-38
Mcromar-Mb-39
Mcromar-Mb-40
Mcromar-Mb-41
Mcromar-Mb-44
Mcromar-Mb-46
Mcromar-Mb-47
Mcromar-Mb-48
Mcromar-Mb-49
Mrugmar-Mb-146
Pgramar-Mb-102
Pgramar-Mb-106
Pgramar-Mb-157
Pgramar-Mb-163
Pgramar-Mb-170
Rmetmar-Mb-2
Rmetmar-Mb-10

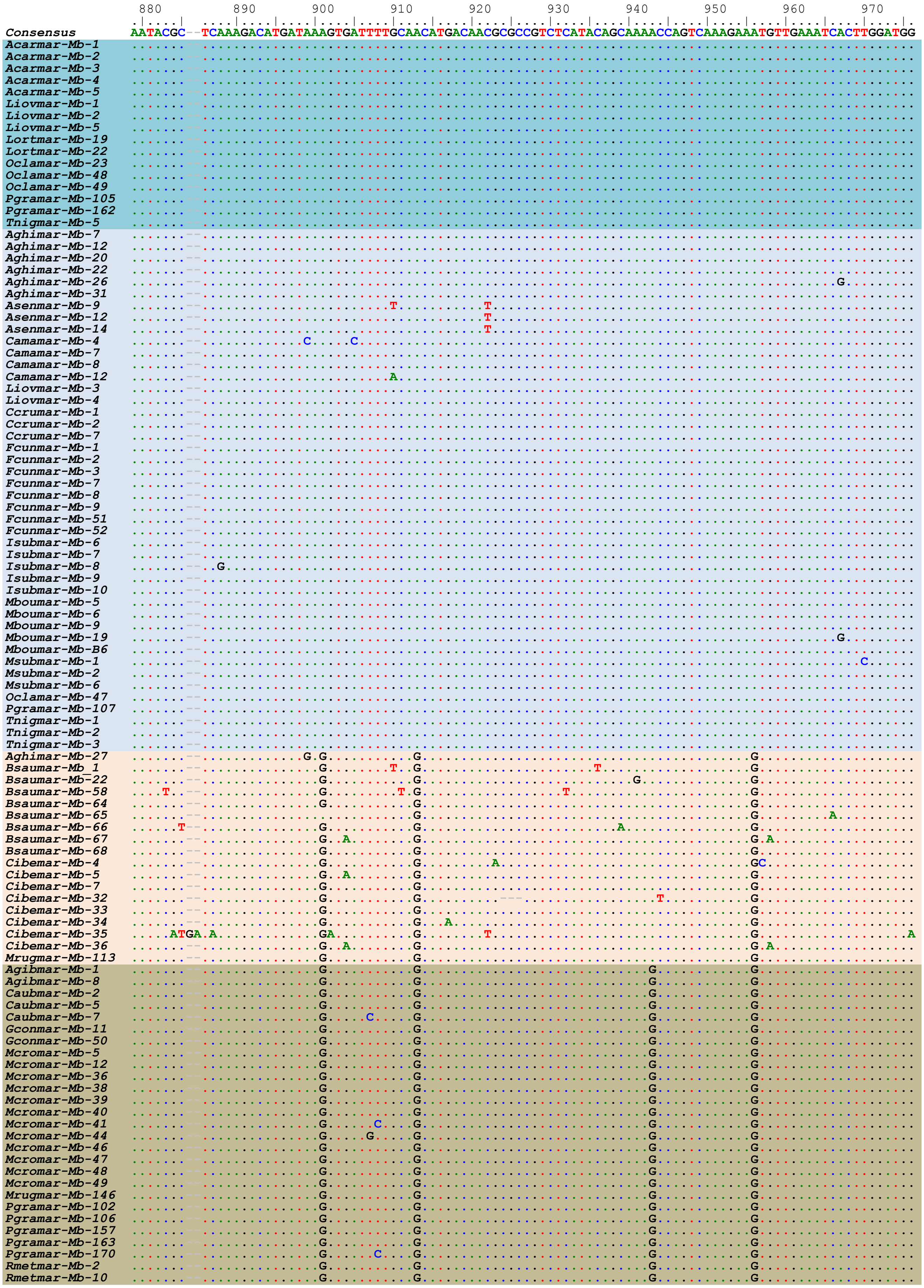

880 890 900 910 920 930 940 950 960 970
Consensus
AATACGC--TCAAAGACATGATAAAGTGATTTTGCAACATGACAACGCGCCGTCTCATACAGCAAAACCAGTCAAAGAAATGTTGAAATCACTTGGATGG
Acarmar-Mb-1
Acarmar-Mb-2
Acarmar-Mb-3
Acarmar-Mb-4
Acarmar-Mb-5
Liovmar-Mb-1
Liovmar-Mb-2
Liovmar-Mb-5
Lortmar-Mb-19
Lortmar-Mb-22
Oclamar-Mb-23
Oclamar-Mb-48
Oclamar-Mb-49
Pgramar-Mb-105
Pgramar-Mb-162
Tnigmar-Mb-5
Aghimar-Mb-7
Aghimar-Mb-12
Aghimar-Mb-20
Aghimar-Mb-22
Aghimar-Mb-26
Aghimar-Mb-31
Asenmar-Mb-9
Asenmar-Mb-12
Asenmar-Mb-14
Camamar-Mb-4
Camamar-Mb-7
Camamar-Mb-8
Camamar-Mb-12
Liovmar-Mb-3
Liovmar-Mb-4
Ccrumar-Mb-1
Ccrumar-Mb-2
Ccrumar-Mb-7
Fcunmar-Mb-1
Fcunmar-Mb-2
Fcunmar-Mb-3
Fcunmar-Mb-7
Fcunmar-Mb-8
Fcunmar-Mb-9
Fcunmar-Mb-51
Fcunmar-Mb-52
Isubmar-Mb-6
Isubmar-Mb-7
Isubmar-Mb-8
Isubmar-Mb-9
Isubmar-Mb-10
Mboumar-Mb-5
Mboumar-Mb-6
Mboumar-Mb-9
Mboumar-Mb-19
Mboumar-Mb-B6
Msubmar-Mb-1
Msubmar-Mb-2
Msubmar-Mb-6
Oclamar-Mb-47
Pgramar-Mb-107
Tnigmar-Mb-1
Tnigmar-Mb-2
Tnigmar-Mb-3
Aghimar-Mb-27
Bsaumar-Mb_1
Bsaumar-Mb-22
Bsaumar-Mb-58
Bsaumar-Mb-64
Bsaumar-Mb-65
Bsaumar-Mb-66
Bsaumar-Mb-67
Bsaumar-Mb-68
Cibemar-Mb-4
Cibemar-Mb-5
Cibemar-Mb-7
Cibemar-Mb-32
Cibemar-Mb-33
Cibemar-Mb-34
Cibemar-Mb-35
Cibemar-Mb-36
Mrugmar-Mb-113
Agibmar-Mb-1
Agibmar-Mb-8
Caubmar-Mb-2
Caubmar-Mb-5
Caubmar-Mb-7
Gconmar-Mb-11
Gconmar-Mb-50
Mcromar-Mb-5
Mcromar-Mb-12
Mcromar-Mb-36
Mcromar-Mb-38
Mcromar-Mb-39
Mcromar-Mb-40
Mcromar-Mb-41
Mcromar-Mb-44
Mcromar-Mb-46
Mcromar-Mb-47
Mcromar-Mb-48
Mcromar-Mb-49
Mrugmar-Mb-146
Pgramar-Mb-102
Pgramar-Mb-106
Pgramar-Mb-157
Pgramar-Mb-163
Pgramar-Mb-170
Rmetmar-Mb-2
Rmetmar-Mb-10

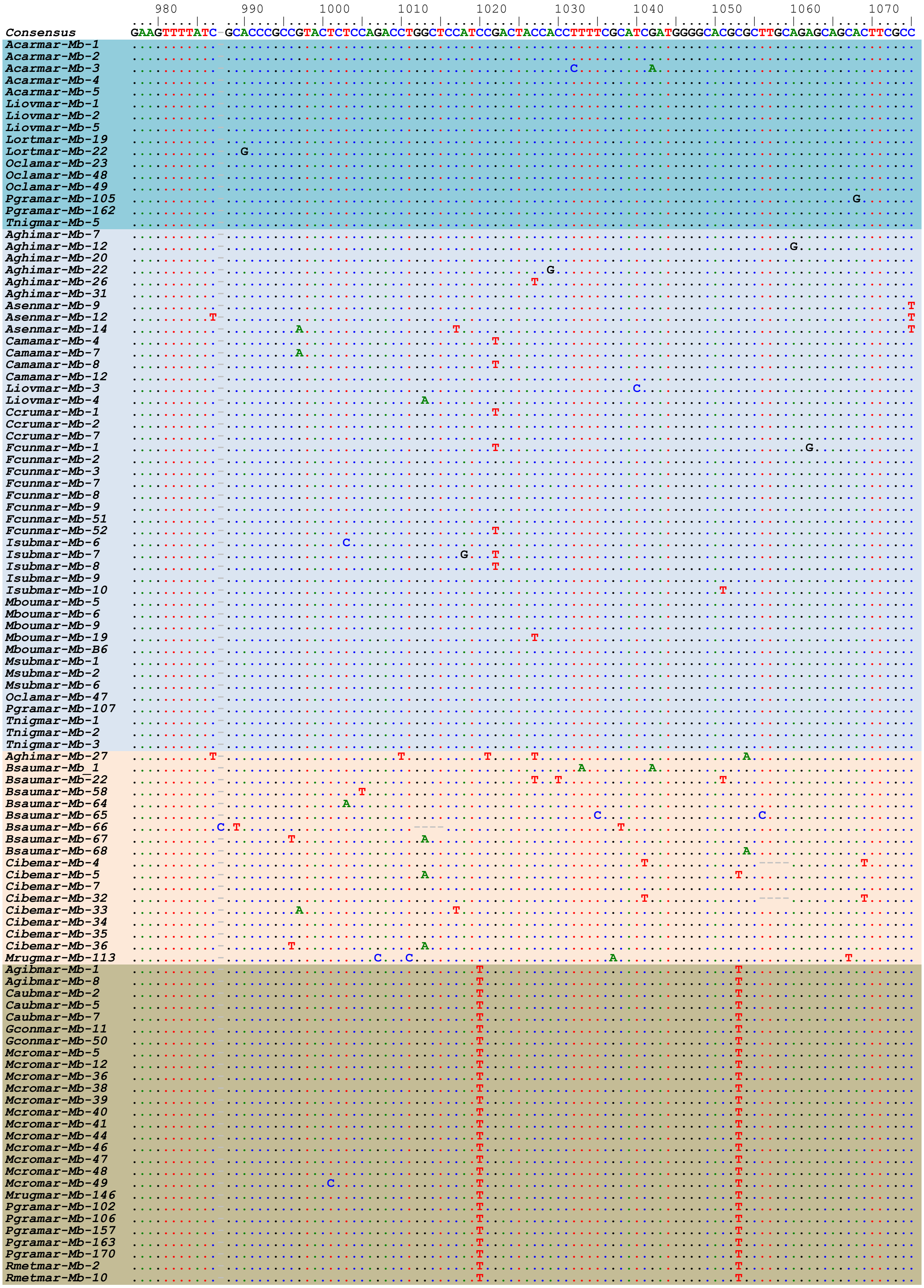
980 990 1000 1010 1020 1030 1040 1050 1060 1070
Consensus GAAGTTTTATC-GCACCCGCCGTACTCTCCAGACCTGGCTCCATCCGACTACCACCTTTTCGCATCGATGGGGCACGCGCTTGCAGAGCAGCACTTCGCC
Acarmar-Mb-1
Acarmar-Mb-2
Acarmar-Mb-3
Acarmar-Mb-4
Acarmar-Mb-5
Liovmar-Mb-1
Liovmar-Mb-2
Liovmar-Mb-5
Lortmar-Mb-19
Lortmar-Mb-22
Oclamar-Mb-23
Oclamar-Mb-48
Oclamar-Mb-49
Pgramar-Mb-105
Pgramar-Mb-162
Tnigmar-Mb-5
Aghimar-Mb-7
Aghimar-Mb-12
Aghimar-Mb-20
Aghimar-Mb-22
Aghimar-Mb-26
Aghimar-Mb-31
Asenmar-Mb-9
Asenmar-Mb-12
Asenmar-Mb-14
Camamar-Mb-4
Camamar-Mb-7
Camamar-Mb-8
Camamar-Mb-12
Liovmar-Mb-3
Liovmar-Mb-4
Ccrumar-Mb-1
Ccrumar-Mb-2
Ccrumar-Mb-7
Fcunmar-Mb-1
Fcunmar-Mb-2
Fcunmar-Mb-3
Fcunmar-Mb-7
Fcunmar-Mb-8
Fcunmar-Mb-9
Fcunmar-Mb-51
Fcunmar-Mb-52
Isubmar-Mb-6
Isubmar-Mb-7
Isubmar-Mb-8
Isubmar-Mb-9
Isubmar-Mb-10
Mboumar-Mb-5
Mboumar-Mb-6
Mboumar-Mb-9
Mboumar-Mb-19
Mboumar-Mb-B6
Msubmar-Mb-1
Msubmar-Mb-2
Msubmar-Mb-6
Oclamar-Mb-47
Pgramar-Mb-107
Tnigmar-Mb-1
Tnigmar-Mb-2
Tnigmar-Mb-3
Aghimar-Mb-27
Bsaumar-Mb_1
Bsaumar-Mb-22
Bsaumar-Mb-58
Bsaumar-Mb-64
Bsaumar-Mb-65
Bsaumar-Mb-66
Bsaumar-Mb-67
Bsaumar-Mb-68
Cibemar-Mb-4
Cibemar-Mb-5
Cibemar-Mb-7
Cibemar-Mb-32
Cibemar-Mb-33
Cibemar-Mb-34
Cibemar-Mb-35
Cibemar-Mb-36
Mrugmar-Mb-113
Agibmar-Mb-1
Agibmar-Mb-8
Caubmar-Mb-2
Caubmar-Mb-5
Caubmar-Mb-7
Gconmar-Mb-11
Gconmar-Mb-50
Mcromar-Mb-5
Mcromar-Mb-12
Mcromar-Mb-36
Mcromar-Mb-38
Mcromar-Mb-39
Mcromar-Mb-40
Mcromar-Mb-41
Mcromar-Mb-44
Mcromar-Mb-46
Mcromar-Mb-47
Mcromar-Mb-48
Mcromar-Mb-49
Mrugmar-Mb-146
Pgramar-Mb-102
Pgramar-Mb-106
Pgramar-Mb-157
Pgramar-Mb-163
Pgramar-Mb-170
Rmetmar-Mb-2
Rmetmar-Mb-10

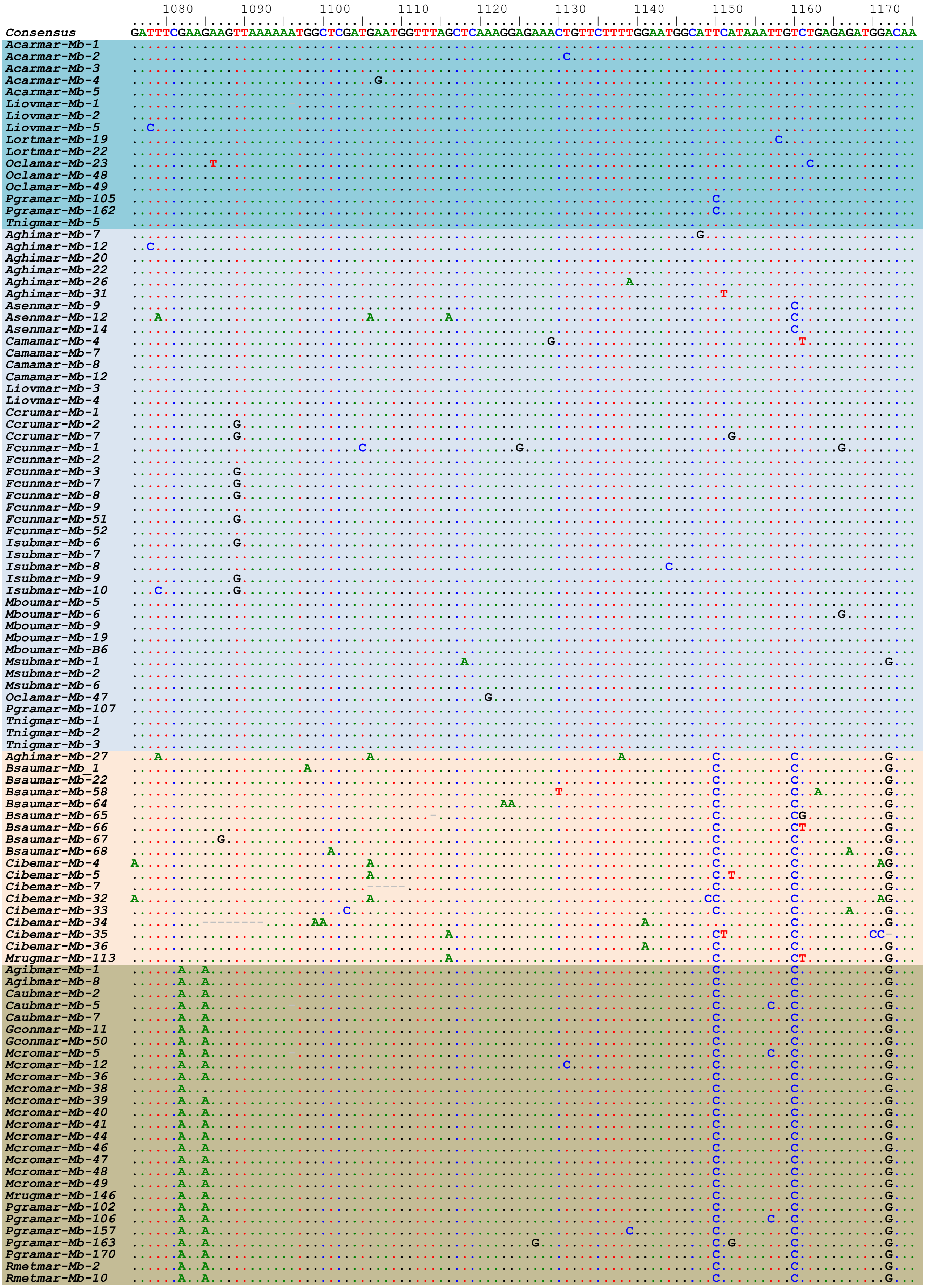
1080 1090 1100 1110 1120 1130 1140 1150 1160 1170
Consensus GATTTCGAAGAAGTTAAAAAATGGCTCGATGAATGGTTTAGCTCAAAGGAGAAACTGTTCTTTTGGAATGGCATTCATAAATTGTCTGAGAGATGGACAA
Acarmar-Mb-1
Acarmar-Mb-2
Acarmar-Mb-3
Acarmar-Mb-4
Acarmar-Mb-5
Liovmar-Mb-1
Liovmar-Mb-2
Liovmar-Mb-5
Lortmar-Mb-19
Lortmar-Mb-22
Oclamar-Mb-23
Oclamar-Mb-48
Oclamar-Mb-49
Pgramar-Mb-105
Pgramar-Mb-162
Tnigmar-Mb-5
Aghimar-Mb-7
Aghimar-Mb-12
Aghimar-Mb-20
Aghimar-Mb-22
Aghimar-Mb-26
Aghimar-Mb-31
Asenmar-Mb-9
Asenmar-Mb-12
Asenmar-Mb-14
Camamar-Mb-4
Camamar-Mb-7
Camamar-Mb-8
Camamar-Mb-12
Liovmar-Mb-3
Liovmar-Mb-4
Ccrumar-Mb-1
Ccrumar-Mb-2
Ccrumar-Mb-7
Fcunmar-Mb-1
Fcunmar-Mb-2
Fcunmar-Mb-3
Fcunmar-Mb-7
Fcunmar-Mb-8
Fcunmar-Mb-9
Fcunmar-Mb-51
Fcunmar-Mb-52
Isubmar-Mb-6
Isubmar-Mb-7
Isubmar-Mb-8
Isubmar-Mb-9
Isubmar-Mb-10
Mboumar-Mb-5
Mboumar-Mb-6
Mboumar-Mb-9
Mboumar-Mb-19
Mboumar-Mb-B6
Msubmar-Mb-1
Msubmar-Mb-2
Msubmar-Mb-6
Oclamar-Mb-47
Pgramar-Mb-107
Tnigmar-Mb-1
Tnigmar-Mb-2
Tnigmar-Mb-3
Aghimar-Mb-27
Bsaumar-Mb_1
Bsaumar-Mb-22
Bsaumar-Mb-58
Bsaumar-Mb-64
Bsaumar-Mb-65
Bsaumar-Mb-66
Bsaumar-Mb-67
Bsaumar-Mb-68
Cibemar-Mb-4
Cibemar-Mb-5
Cibemar-Mb-7
Cibemar-Mb-32
Cibemar-Mb-33
Cibemar-Mb-34
Cibemar-Mb-35
Cibemar-Mb-36
Mrugmar-Mb-113
Agibmar-Mb-1
Agibmar-Mb-8
Caubmar-Mb-2
Caubmar-Mb-5
Caubmar-Mb-7
Gconmar-Mb-11
Gconmar-Mb-50
Mcromar-Mb-5
Mcromar-Mb-12
Mcromar-Mb-36
Mcromar-Mb-38
Mcromar-Mb-39
Mcromar-Mb-40
Mcromar-Mb-41
Mcromar-Mb-44
Mcromar-Mb-46
Mcromar-Mb-47
Mcromar-Mb-48
Mcromar-Mb-49
Mrugmar-Mb-146
Pgramar-Mb-102
Pgramar-Mb-106
Pgramar-Mb-157
Pgramar-Mb-163
Pgramar-Mb-170
Rmetmar-Mb-2
Rmetmar-Mb-10

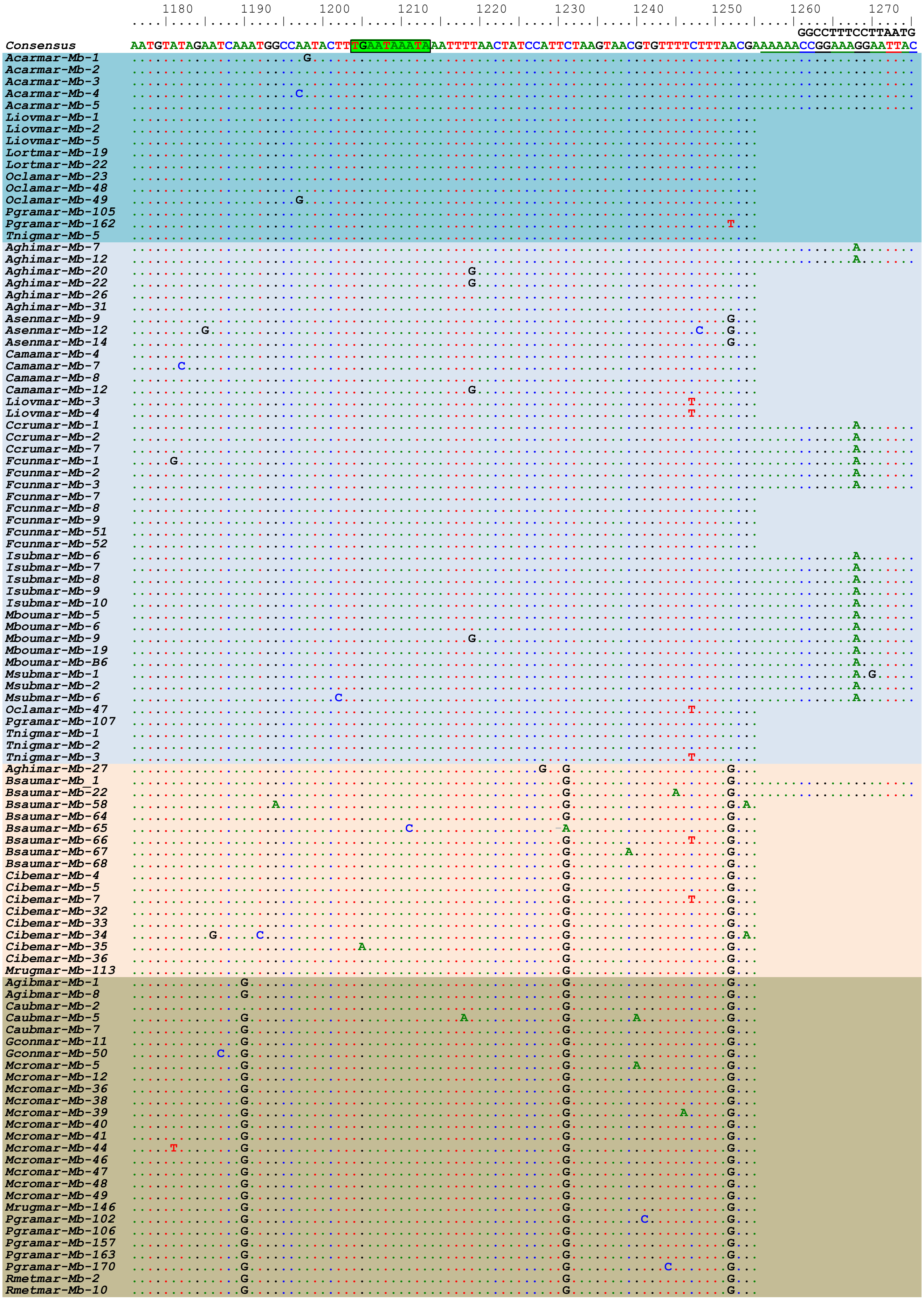

1180 1190 1200 1210 1220 1230 1240 1250 1260 1270
GGCCTTTCCTTAATG
Consensus AATGTATAGAATCAAATGGCCAATACTTTGAATAAATAAATTTTAACTATCCATTCTAAGTAACGTGTTTTCTTTAACGAAAAAACCGGAAAGGAATTAC
Acarmar-Mb-1
Acarmar-Mb-2
Acarmar-Mb-3
Acarmar-Mb-4
Acarmar-Mb-5
Liovmar-Mb-1
Liovmar-Mb-2
Liovmar-Mb-5
Lortmar-Mb-19
Lortmar-Mb-22
Oclamar-Mb-23
Oclamar-Mb-48
Oclamar-Mb-49
Pgramar-Mb-105
Pgramar-Mb-162
Tnigmar-Mb-5
Aghimar-Mb-7
Aghimar-Mb-12
Aghimar-Mb-20
Aghimar-Mb-22
Aghimar-Mb-26
Aghimar-Mb-31
Asenmar-Mb-9
Asenmar-Mb-12
Asenmar-Mb-14
Camamar-Mb-4
Camamar-Mb-7
Camamar-Mb-8
Camamar-Mb-12
Liovmar-Mb-3
Liovmar-Mb-4
Ccrumar-Mb-1
Ccrumar-Mb-2
Ccrumar-Mb-7
Fcunmar-Mb-1
Fcunmar-Mb-2
Fcunmar-Mb-3
Fcunmar-Mb-7
Fcunmar-Mb-8
Fcunmar-Mb-9
Fcunmar-Mb-51
Fcunmar-Mb-52
Isubmar-Mb-6
Isubmar-Mb-7
Isubmar-Mb-8
Isubmar-Mb-9
Isubmar-Mb-10
Mboumar-Mb-5
Mboumar-Mb-6
Mboumar-Mb-9
Mboumar-Mb-19
Mboumar-Mb-B6
Msubmar-Mb-1
Msubmar-Mb-2
Msubmar-Mb-6
Oclamar-Mb-47
Pgramar-Mb-107
Tnigmar-Mb-1
Tnigmar-Mb-2
Tnigmar-Mb-3
Aghimar-Mb-27
Bsaumar-Mb_1
Bsaumar-Mb-22
Bsaumar-Mb-58
Bsaumar-Mb-64
Bsaumar-Mb-65
Bsaumar-Mb-66
Bsaumar-Mb-67
Bsaumar-Mb-68
Cibemar-Mb-4
Cibemar-Mb-5
Cibemar-Mb-7
Cibemar-Mb-32
Cibemar-Mb-33
Cibemar-Mb-34
Cibemar-Mb-35
Cibemar-Mb-36
Mrugmar-Mb-113
Agibmar-Mb-1
Agibmar-Mb-8
Caubmar-Mb-2
Caubmar-Mb-5
Caubmar-Mb-7
Gconmar-Mb-11
Gconmar-Mb-50
Mcromar-Mb-5
Mcromar-Mb-12
Mcromar-Mb-36
Mcromar-Mb-38
Mcromar-Mb-39
Mcromar-Mb-40
Mcromar-Mb-41
Mcromar-Mb-44
Mcromar-Mb-46
Mcromar-Mb-47
Mcromar-Mb-48
Mcromar-Mb-49
Mrugmar-Mb-146
Pgramar-Mb-102
Pgramar-Mb-106
Pgramar-Mb-157
Pgramar-Mb-163
Pgramar-Mb-170
Rmetmar-Mb-2
Rmetmar-Mb-10

	1280	
	....|....|..	
	GCTGTGTGGACC- 5'	
Consensus	CGACACACCTGG	
Acarmar-Mb-1	....	1287
Acarmar-Mb-2	....	1287
Acarmar-Mb-3	....	1287
Acarmar-Mb-4	....	1287
Acarmar-Mb-5	....	1287
Liovmar-Mb-1		1286
Liovmar-Mb-2		1287
Liovmar-Mb-5		1287
Lortmar-Mb-19		1287
Lortmar-Mb-22		1287
Oclamar-Mb-23		1287
Oclamar-Mb-48		1287
Oclamar-Mb-49		1289
Pgramar-Mb-105		1287
Pgramar-Mb-162		1287
Tnigmar-Mb-5		1287
Aghimar-Mb-7	....	1286
Aghimar-Mb-12	....	1286
Aghimar-Mb-20		1287
Aghimar-Mb-22		1287
Aghimar-Mb-26		1287
Aghimar-Mb-31		1286
Asenmar-Mb-9		1279
Asenmar-Mb-12		1287
Asenmar-Mb-14		1287
Camamar-Mb-4		1286
Camamar-Mb-7		1287
Camamar-Mb-8		1287
Camamar-Mb-12		1287
Liovmar-Mb-3		1287
Liovmar-Mb-4		1287
Ccrumar-Mb-1	....	1287
Ccrumar-Mb-2	....	1287
Ccrumar-Mb-7	....	1286
Fcunmar-Mb-1	....	1287
Fcunmar-Mb-2	....	1287
Fcunmar-Mb-3	.........C..	1287
Fcunmar-Mb-7		1287
Fcunmar-Mb-8		1287
Fcunmar-Mb-9		1287
Fcunmar-Mb-51		1287
Fcunmar-Mb-52		1287
Isubmar-Mb-6	....	1287
Isubmar-Mb-7	....	1286
Isubmar-Mb-8	....	1287
Isubmar-Mb-9	....	1287
Isubmar-Mb-10	....	1286
Mboumar-Mb-5	....	1286
Mboumar-Mb-6	....	1287
Mboumar-Mb-9	......T.....	1287
Mboumar-Mb-19	....	1287
Mboumar-Mb-B6	....	1287
Msubmar-Mb-1	....	1287
Msubmar-Mb-2	....	1287
Msubmar-Mb-6	....	1286
Oclamar-Mb-47		1287
Pgramar-Mb-107		1286
Tnigmar-Mb-1		1286
Tnigmar-Mb-2		1287
Tnigmar-Mb-3		1287
Aghimar-Mb-27		1287
Bsaumar-Mb_1	....	1284
Bsaumar-Mb-22	....	1151
Bsaumar-Mb-58		1248
Bsaumar-Mb-64		1153
Bsaumar-Mb-65		1285
Bsaumar-Mb-66		1277
Bsaumar-Mb-67		1290
Bsaumar-Mb-68		1283
Cibemar-Mb-4		1284
Cibemar-Mb-5		1279
Cibemar-Mb-7		1295
Cibemar-Mb-32		1280
Cibemar-Mb-33		1287
Cibemar-Mb-34		1277
Cibemar-Mb-35		1288
Cibemar-Mb-36		1285
Mrugmar-Mb-113		1287
Agibmar-Mb-1		1280
Agibmar-Mb-8		1280
Caubmar-Mb-2		1280
Caubmar-Mb-5		1279
Caubmar-Mb-7		1280
Gconmar-Mb-11		1279
Gconmar-Mb-50		1280
Mcromar-Mb-5		1279
Mcromar-Mb-12		1279
Mcromar-Mb-36		1280
Mcromar-Mb-38		1280
Mcromar-Mb-39		1280
Mcromar-Mb-40		1280
Mcromar-Mb-41		1280
Mcromar-Mb-44		1280
Mcromar-Mb-46		1280
Mcromar-Mb-47		1280
Mcromar-Mb-48		1280
Mcromar-Mb-49		1280
Mrugmar-Mb-146		1279
Pgramar-Mb-102		1280
Pgramar-Mb-106		1280
Pgramar-Mb-157		1280
Pgramar-Mb-163		1280
Pgramar-Mb-170		1280
Rmetmar-Mb-2		1280
Rmetmar-Mb-10		1280

Figure S2. Sequence alignment of all putative transposases in relation to the Mboumar-9 transposase (Palomeque et al. 2006). The conserved D,D(34)D motif, the bipartite nuclear location signal (NLS), and the helix-turn-helix (HTH) motif are shown.

```
                                                                                                     HTH motif
Mboumar-Mb-9     MSSFVPENVHLRHALLFLFHQKKRAAESHRLLVETYGEHAPTIRTCETWFRQFKCGDFNVQDKERPGRPKTFEDAELQELLDEDSTQTQKQLAEKLNVSR 100
Aghimar-Mb-20    ....................................................................................................
Aghimar-Mb-22    ........A...........................................................................................
Aghimar-Mb-26    .......................K............................................L...............................
Aghimar-Mb-27    .......................K..........................Q...Y..........L..................P...............
Asenmar-Mb-9     .......................K.....................................................................K......
Asenmar-Mb-12    .......................K............................................................P...............
Camamar-Mb-8     .......................KV...R.......................................................................
Camamar-Mb-12    .......................K........................................................................S...
Ccrumar-Mb-1     .......................K............................................................................
Liovmar-Mb-3     .......................K............................................................................
Liovmar-Mb-4     .......................K.......................A....................................................
Fcunmar-Mb-2     .......................K............................................................................
Fcunmar-Mb-3     .......................K..........................................................................G.
Fcunmar-Mb-7     .......................K............................................................................
Fcunmar-Mb-8     .......................K............................................................................
Fcunmar-Mb-51    .................S.....K...................K........................................................
Fcunmar-Mb-52    ...............F.......K..........I..............................S..................................
Isubmar-Mb-7     .......................K.........G..................................................................
Isubmar-Mb-8     .......................K............................................................................
Mboumar-Mb-6     .......................K............................................L...............................
Mboumar-Mb-19    .......................K............................................L...............................
Mboumar-Mb-B6    .......................K............................................................................
Msubmar-Mb-1     .......................K...................K........................................................
Msubmar-Mb-2     .......................K...................K........................................................
Oclamar-Mb-47    .......................K............................................................................
Tnigmar-Mb-1     .......................K............................................................................
Tnigmar-Mb-2     .......................K............................................................................
Tnigmar-Mb-3     .......................K............................................................................

                                 Conserved       Bipartite
                                    Motif     nuclear location signal    D
Mboumar-Mb-9     VAICERLQAMGKIQKMGRWVPHELNDRQMENRKIVSEMLLQRYERKSFLHRIVTGDEKWIYFENPKRKKSWLSPGEAGPSTARPNRFGRKTMLCVWWDQI 200
Aghimar-Mb-20    ....................................................................................................
Aghimar-Mb-22    ....................................................................................................
Aghimar-Mb-26    ........................................................G...........................................
Aghimar-Mb-27    ............................L...E................................................T..................
Asenmar-Mb-9     ............................I.................................................L.....................
Asenmar-Mb-12    ................................................................H.............L.....................
Camamar-Mb-8     ....................S..................F......................................S.....................
Camamar-Mb-12    ...........................................................M........................................
Ccrumar-Mb-1     ....................................................................................................
Liovmar-Mb-3     ....................................................................................................
Liovmar-Mb-4     ....................................................................................................
Fcunmar-Mb-2     ..........................................H.........................................................
Fcunmar-Mb-3     ....................................................................................................
Fcunmar-Mb-7     .....................................I............................................K.........P.......
Fcunmar-Mb-8     ....................................................................................................
Fcunmar-Mb-51    ............................................................................V.......................
Fcunmar-Mb-52    ....................................................................................................
Isubmar-Mb-7     ..............................................P.....................................................
Isubmar-Mb-8     ....................................................................................................
Mboumar-Mb-6     ....................................................................................................
Mboumar-Mb-19    ........................................................G...........................................
Mboumar-Mb-B6    .......................................................G............................................
Msubmar-Mb-1     .....Q..............................................................................................
Msubmar-Mb-2     ....................................................................................................
Oclamar-Mb-47    ...Y........V..........................P.................E..........................................
Tnigmar-Mb-1     ....................................................................................................
Tnigmar-Mb-2     ....................................................................................................
Tnigmar-Mb-3     ....................................................................................................

                                                                                           Conserved
                                                    D                                        motif   D
Mboumar-Mb-9     GVVYYELLKPGETVNTDRYRQQMINLNCALIEKRPQYAQRHDKVILQHDNAPSHTAKPVKEMLKSLGWEVLSHPPYSPDLAPSDYHLFASMGHALAEQHF 300
Aghimar-Mb-20    ....................................................................................................
Aghimar-Mb-22    .....................................................................................R..............
Aghimar-Mb-26    ........................S...........................................................................
Aghimar-Mb-27    ..........R...........V....................E..................V.........L............................
Asenmar-Mb-9     ...........................G.................F......................................................
Asenmar-Mb-12    ...........................G.....................................................L..................
Camamar-Mb-8     ...................................................................................Y................
Camamar-Mb-12    ....................................................................................................
Ccrumar-Mb-1     ........................D..........................................................Y................
Liovmar-Mb-3     .........................................................................................P..........
Liovmar-Mb-4     ................................................................................T...................
Fcunmar-Mb-2     ....................................................................................................
Fcunmar-Mb-3     ....................................................................................................
Fcunmar-Mb-7     ....................................................................................................
Fcunmar-Mb-8     ....................................................................................................
Fcunmar-Mb-51    ....................................................................................................
Fcunmar-Mb-52    ...................................................................................Y................
Isubmar-Mb-7     ...................................................................................Y................
Isubmar-Mb-8     ......................................R............................................Y................
Mboumar-Mb-6     ....................................................................................................
Mboumar-Mb-19    ....................................................................................................
Mboumar-Mb-B6    ....................................................................................................
Msubmar-Mb-1     ....................................................................................................
Msubmar-Mb-2     ....................................................................................................
```

```
Oclamar-Mb-47    ....................................................................................................
Tnigmar-Mb-1     ...........................Y........................................................................
Tnigmar-Mb-2     ................................R...................................................................
Tnigmar-Mb-3     ....................................................................................................

Mboumar-Mb-9     ADFEEVKKWLDEWFSSKEKLFFWNGIHKLSERWTKCIESNGQYFE 345
Aghimar-Mb-20    .............................................
Aghimar-Mb-22    .............................................
Aghimar-Mb-26    ......................R......................
Aghimar-Mb-27    ..I........K.........L.......P...A...........
Asenmar-Mb-9     .............................P...............
Asenmar-Mb-12    .............................P...............
Camamar-Mb-8     .............................................
Camamar-Mb-12    .............................................
Ccrumar-Mb-1     .............................................
Liovmar-Mb-3     .............................................
Liovmar-Mb-4     .............................................
Fcunmar-Mb-2     .............................................
Fcunmar-Mb-3     .....G.......................................
Fcunmar-Mb-7     .....G.......................................
Fcunmar-Mb-8     .....G.......................................
Fcunmar-Mb-51    .....G.......................................
Fcunmar-Mb-52    .............................................
Isubmar-Mb-7     .............................................
Isubmar-Mb-8     .............................................
Mboumar-Mb-6     ...............................G.............
Mboumar-Mb-19    .............................................
Mboumar-Mb-B6    .............................................
Msubmar-Mb-1     ...............T.................A...........
Msubmar-Mb-2     .............................................
Oclamar-Mb-47    ................E............................
Tnigmar-Mb-1     .............................................
Tnigmar-Mb-2     .............................................
Tnigmar-Mb-3     .............................................
```

Figure S3. Timetree of 213 ant genera obtained from the timetree.org website. In this tree, we have indicated with "+" or "-" those genera in which it has been possible or not possible to find the *Mb mariner* elements. The numbers indicate the number of species with positive or negative results.

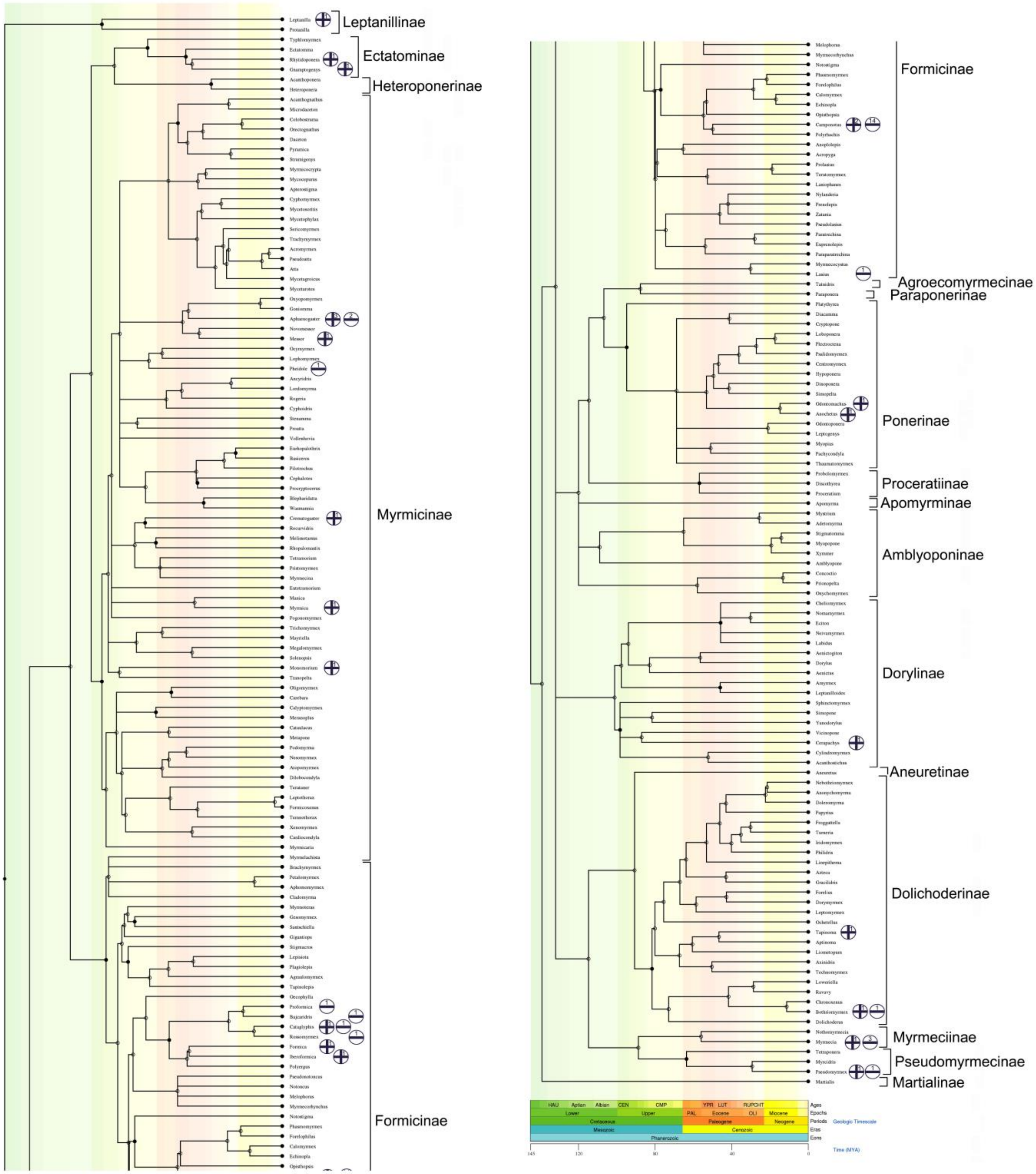

Table S1. Sampling location of analysed ants and GenBank accession numbers of *wingless* (*wnt-1*), *abdominal-A* (*abdA*), and *long-wavelength rhodopsin* (*lwRh*) sequences used in Figure 2. GenBank accession numbers of *mariner* elements isolated in each species are also shown. The shaded sequences were retrieved directly from GenBank. (***) Sequences that we have been unable to amplify after repeated attempts. For the analyses, these sequences were replaced by a consensus sequence obtained for other species of the same genus that were available in GenBank. The remaining sequences have been obtained by the authors.

Species	Code	Location	*wingless*	*abdominal*	*rodopsina*	*mariner*
Aphaenogaster cardenai	FU-1	Constantina, Sevilla (Spain)	LT623141	LT623154	LT623148	LT632334-LT632338
Aphaenogaster gibbosa	CA-44	Canena, Jaén (Spain)	LT623143	LT623156	LT623150	LT632339-LT632340
Aphaenogaster senilis	RF-417	Rio Frio, Jaén (Spain)	LT623145	FJ824329	EF519068	LT632341-LT632343
Anochetus ghilianii	AG-1	Algeciras, Cadiz (Spain)	LT623160	LT623172	LT632323	LT632344-LT632350
Bothriomyrmex saundersi	SDR-1	Sierra del Rey, Málaga (Spain)	FJ940021	FJ939817	FJ939988	LT632351-LT632358
Camponotus amaurus	AB-3	Abla, Almería (Spain)	LT623161	LT623173	LT632324	LT632359-LT632362
Camponotus cruentatus	MF-1	Montefrío, Granada (Spain)	LT623162	LT623174	LT632325	LT632363-LT632365
Cataglyphis iberica	CG-53	Cabo de Gata, Almería (Spain)	DQ353077	DQ352909	DQ353194	LT632366-LT632373
Crematogaster auberti	FT-1	Fuente Teja, Sierra de Huetor, Granada (Spain)	LT623164	LT623176	LT632327	LT632379-LT632381
Formica cunicularia	CU-112	Campus Universidad Jaén (Spain)	LT623165	LT623177	LT632328	LT632382-LT632389
Gnamptogenys continua	BR-3	Serra Bonita (Brazil)	LT623166	***	LT632329	LT632390-LT632391
Iberoformica subrufa	CA-35	Canena, Jaén (Spain)	LT623167	LT623178	LT632330	LT632392-LT632396
Leptanilla ortunoi	CE-1	Ceuta (Spain)	LT623168	LT623179	LT632331	LT632397-LT632398
Lioponera iovis	AUT-3	Springrange (Australia)	LT623163	LT623175	LT632326	LT632374-LT632378
Messor bouvieri	CA-15	Canena, Jaén (Spain)	HE963097	LT623180	HE963100	AJ781768-AJ781772
Monomorium subopacum	GR-10	Motril, Granada (Spain)	LT623169	***	***	LT632399-LT632401
Myrmecia croslandi	AUT-2	Corang River Bridge (Australia)	LT623170	LT623181	LT632332	LT632402-LT632413
Myrmica ruginodis	TO-3	Tours (France)	HE963098	FJ824349	HE963101, LT632333	LT632414-LT632415
Odontomachus clarus	MEJ-2	Puerto Vallarta (Mexico)	EU155470	DQ352977	EU155451	LT632416-LT632419
Pseudomyrmex gracillis	URU-1	Montevideo (Uruguay)	FJ436851	AY703730	AY703797	LT632420-LT632427
Rhytidoponera metallica	AUT-4	Camberra (Australia)	DQ353097	DQ352926	DQ353239	LT632428-LT632429
Tapinoma ibericum	CV-6	Los Villares, Jaén (Spain)	HE963099	HE963096	HE963102	HE577149-HE577152

Table S2. Estimates of evolutionary divergence within *mariner* element sequences isolated from the same species. Analyses were conducted using the Tamura 3-parameter model. The rate variation among sites was modeled with a gamma distribution (shape parameter = 2.5). Standard error estimates are shown in the third column.

	d	*S.E.*
Acarmar-Mb	0.004	0.001
Agibmar-Mb	0.002	0.001
Aghimar-Mb	0.017	0.002
Asenmar-Mb	0.014	0.003
Bsaumar-Mb	0.030	0.003
Camamar-Mb	0.012	0.002
Caubmar-Mb	0.004	0.002
Cibermar-Mb	0.032	0.003
Ccrumar-Mb	0.008	0.002
Fcunmar-Mb	0.008	0.001
Gconmar-Mb	0.003	0.002
Isubmar-Mb	0.011	0.002
Liovmar-Mb	0.008	0.002
Lortmar-Mb	0.008	0.003
Mboumar-Mb	0.004	0.001
Mcromar-Mb	0.003	0.001
Mrugmar-Mb	0.036	0.005
Msubmar-Mb	0.008	0.002
Oclamar-Mb	0.008	0.002
Pgramar-Mb	0.027	0.003
Rmetmar-Mb	0.001	0.001
Tnigmar-Mb	0.004	0.001

Table S3. Estimates of evolutionary divergence between *mariner* elements isolated from different species. Analyses were conducted using the Tamura 3-parameter model. The rate variation among sites was modeled with a gamma distribution (shape parameter = 2.5). Standard error estimates are shown above the diagonal.

	(1)	(2)	(3)	(4)	(5)	(6)	(7)	(8)	(9)	(10)	(11)	(12)	(13)	(14)	(15)	(16)	(17)	(18)	(19)	(20)	(21)	(22)
(1) *Acamar-Mb*		0.002	0.006	0.003	0.005	0.002	0.006	0.005	0.002	0.002	0.006	0.002	0.001	0.002	0.002	0.006	0.005	0.002	0.001	0.004	0.006	0.001
(2) *Agibmar-Mb*	0.014		0.005	0.003	0.004	0.002	0.005	0.004	0.002	0.001	0.005	0.002	0.002	0.003	0.001	0.005	0.004	0.002	0.002	0.004	0.005	0.001
(3) *Aghimar-Mb*	0.045	0.043		0.006	0.004	0.006	0.001	0.004	0.006	0.006	0.001	0.006	0.006	0.006	0.006	0.001	0.003	0.006	0.006	0.002	0.001	0.006
(4) *Asenmar-Mb*	0.018	0.020	0.047		0.004	0.003	0.006	0.004	0.003	0.003	0.006	0.003	0.003	0.003	0.003	0.006	0.005	0.003	0.003	0.004	0.006	0.003
(5) *Bsaumar-Mb*	0.039	0.037	0.036	0.041		0.004	0.004	0.002	0.004	0.004	0.004	0.004	0.004	0.005	0.004	0.004	0.003	0.004	0.004	0.003	0.004	0.004
(6) *Camamar-Mb*	0.011	0.014	0.045	0.018	0.040		0.006	0.004	0.002	0.001	0.006	0.002	0.002	0.003	0.001	0.006	0.005	0.002	0.002	0.004	0.006	0.001
(7) *Caubmar-Mb*	0.045	0.044	0.003	0.048	0.037	0.046		0.004	0.006	0.006	0.001	0.006	0.006	0.006	0.006	0.001	0.003	0.006	0.006	0.002	0.001	0.006
(8) *Cibermar-Mb*	0.040	0.038	0.036	0.041	0.031	0.040	0.037		0.004	0.004	0.004	0.004	0.004	0.005	0.004	0.004	0.003	0.004	0.004	0.003	0.004	0.004
(9) *Ccrumar-Mb*	0.011	0.014	0.044	0.018	0.039	0.011	0.045	0.040		0.001	0.006	0.001	0.002	0.003	0.001	0.006	0.005	0.002	0.002	0.004	0.006	0.002
(10) *Fcunmar-Mb*	0.010	0.013	0.043	0.017	0.038	0.010	0.044	0.039	0.008		0.006	0.001	0.001	0.003	0.001	0.006	0.005	0.001	0.002	0.004	0.006	0.001
(11) *Gconmar-Mb*	0.045	0.044	0.002	0.048	0.037	0.046	0.004	0.037	0.045	0.044		0.006	0.006	0.006	0.006	0.001	0.003	0.006	0.006	0.002	0.001	0.006
(12) *Isubmar-Mb*	0.012	0.015	0.046	0.019	0.041	0.012	0.046	0.041	0.009	0.009	0.046		0.002	0.003	0.001	0.006	0.005	0.001	0.002	0.004	0.006	0.001
(13) *Liovmar-Mb*	0.007	0.014	0.044	0.018	0.039	0.011	0.045	0.039	0.010	0.009	0.045	0.012		0.002	0.002	0.006	0.005	0.002	0.001	0.004	0.006	0.001
(14) *Lortmar-Mb*	0.008	0.018	0.049	0.022	0.044	0.016	0.050	0.044	0.015	0.014	0.050	0.016	0.011		0.003	0.006	0.006	0.003	0.002	0.004	0.006	0.002
(15) *Mboumar-Mb*	0.008	0.010	0.041	0.016	0.036	0.009	0.042	0.037	0.007	0.007	0.042	0.008	0.008	0.012		0.006	0.005	0.001	0.002	0.004	0.006	0.001
(16) *Mcromar-Mb*	0.045	0.043	0.002	0.048	0.037	0.046	0.004	0.037	0.045	0.044	0.003	0.046	0.045	0.050	0.042		0.003	0.006	0.006	0.002	0.001	0.006
(17) *Mrugmar-Mb*	0.043	0.041	0.019	0.045	0.034	0.043	0.020	0.034	0.043	0.041	0.020	0.044	0.042	0.047	0.040	0.020		0.005	0.005	0.003	0.003	0.005
(18) *Msubmar-Mb*	0.010	0.013	0.044	0.018	0.038	0.010	0.044	0.039	0.009	0.008	0.044	0.009	0.010	0.015	0.006	0.044	0.042		0.002	0.004	0.006	0.001
(19) *Oclamar-Mb*	0.006	0.014	0.045	0.018	0.039	0.011	0.045	0.040	0.011	0.010	0.045	0.012	0.007	0.010	0.008	0.045	0.043	0.010		0.004	0.006	0.001
(20) *Pgramar-Mb*	0.031	0.033	0.018	0.037	0.038	0.034	0.019	0.038	0.033	0.032	0.019	0.034	0.032	0.036	0.030	0.019	0.028	0.032	0.032		0.002	0.004
(21) *Rmetmar-Mb*	0.044	0.042	0.001	0.046	0.036	0.045	0.002	0.036	0.044	0.043	0.002	0.045	0.043	0.049	0.041	0.002	0.018	0.043	0.044	0.018		0.006
(22)*Tnigmar-Mb*	0.006	0.011	0.041	0.015	0.035	0.008	0.041	0.036	0.007	0.007	0.041	0.009	0.006	0.010	0.005	0.041	0.039	0.007	0.006	0.029	0.040	

Table S4. Estimates of evolutionary divergence within and between the considered groups in *Mboumar*-like *mariner* sequences. The number of fixed differences between them is shown in parentheses.

	Clade I	Subclade II-1	Rest of clade II
Clade I	0.005±0.001		
Subclade II-1	0.046±0.006 (46)	0.003±0.001	
Remaining sequences of Clade II	0.040±0.004 (16)	0.037±0.004 (13)	0.031±0.002

Table S5. Codon-based Test of Purifying Selection for analysis averaging over all sequence pairs and within each group of sequences. The probability of rejecting the null hypothesis of strict-neutrality (dN=dS) in favor of the alternative hypothesis (dN<dS) is shown in the column called Prob. Values of P lower than 0.05 are considered significant at the 5% level. The statistical test (dS-dN) is shown in the column called Stat. dN and dS are the number of synonymous and nonsynonymous substitutions per site, respectively. The variance of the difference was computed using the bootstrap method (1000 replicates). dN/dS are shown in the first column.

	dN/dS	Prob	Stat
All sequences	0.454	0.003	2.831
Clade I	0.619	0.036	1.816
Clade II	0.387	0.001	3.200
Subclade II-1	0.292	0.005	2.601
Remaining sequences of Clade II	0.627	0.006	2.570

Table S6. Estimates of the mean synonymous divergence (K_S) between ant species. These values were obtained using the sequences of the three loci from each species (*abdA*, *lw-Rh*, and *wnt-1*).

	(1)	(2)	(3)	(4)	(5)	(6)	(7)	(8)	(9)	(10)	(11)	(12)	(13)	(14)	(15)	(16)	(17)	(18)	(19)	(20)	(21)	(22)
(1) *Anochetus ghillianii*																						
(2) *Aphaenogaster cardenai*	0.51																					
(3) *Aphaenogaster gibbosa*	0.55	0.07																				
(4) *Aphaenogaster senilis*	0.60	0.08	0.06																			
(5) *Bothriomyrmexsaundersii*	0.64	0.50	0.50	0.49																		
(6) *Camponotus amaurus*	0.64	0.53	0.55	0.58	0.56																	
(7) *Camponotus cruentatus*	0.64	0.53	0.55	0.57	0.56	0.03																
(8) *Cataglyphis iberica*	0.77	0.57	0.61	0.59	0.56	0.41	0.42															
(9) *Crematogaster auberti*	0.60	0.30	0.33	0.35	0.57	0.55	0.54	0.61														
(10) *Formica cunicularia*	0.59	0.55	0.61	0.60	0.57	0.38	0.37	0.17	0.63													
(11) *Gnamptogenys continua*	0.50	0.38	0.40	0.44	0.43	0.48	0.47	0.53	0.42	0.52												
(12) *Iberoformica subrufa*	0.61	0.56	0.63	0.61	0.60	0.42	0.40	0.21	0.63	0.07	0.56											
(13) *Leptanilla ortunoi*	1.37	1.26	1.26	1.24	1.19	1.15	1.19	1.14	1.19	1.21	1.07	1.21										
(14) *Lioponera iovis*	0.60	0.57	0.62	0.62	0.60	0.62	0.63	0.64	0.59	0.59	0.47	0.62	1.12									
(15) *Messor bouvieri*	0.58	0.07	0.07	0.08	0.50	0.52	0.53	0.57	0.31	0.62	0.40	0.64	1.22	0.58								
(16) *Monomorium subopacum*	0.59	0.32	0.33	0.35	0.58	0.55	0.58	0.68	0.36	0.62	0.45	0.65	1.25	0.58	0.33							
(17) *Myrmecia croslandi*	0.68	0.62	0.62	0.62	0.59	0.72	0.72	0.63	0.60	0.68	0.42	0.69	1.31	0.63	0.62	0.64						
(18) *Myrmica ruginodis*	0.51	0.23	0.25	0.25	0.47	0.52	0.52	0.55	0.33	0.49	0.33	0.52	1.25	0.55	0.27	0.36	0.61					
(19) *Odontomachus clarus*	0.14	0.53	0.55	0.59	0.62	0.69	0.68	0.73	0.58	0.65	0.51	0.66	1.39	0.63	0.58	0.61	0.67	0.52				
(20) *Pseudomyrmex gracillis*	0.78	0.73	0.74	0.77	0.76	0.95	0.91	0.98	0.70	0.92	0.64	1.00	1.40	0.79	0.77	0.78	0.72	0.64	0.82			
(21) *Rhytidoponera metallica*	0.52	0.37	0.39	0.42	0.40	0.50	0.52	0.56	0.46	0.55	0.20	0.55	1.05	0.44	0.41	0.45	0.47	0.35	0.56	0.57		
(22) *Tapinoma ibericum*	0.59	0.46	0.50	0.48	0.27	0.57	0.57	0.58	0.58	0.57	0.40	0.61	1.27	0.57	0.51	0.46	0.57	0.44	0.61	0.70	0.41	

Table S7. Estimates of the mean synonymous divergence (K_S) between *Mboumar*-like *mariner* elements. Average K_S values and their standard errors are indicated below and above the diagonal, respectively.

	(1)	(2)	(3)	(4)	(5)	(6)	(7)	(8)	(9)	(10)	(11)	(12)	(13)	(14)	(15)	(16)	(17)	(18)	(19)	(20)	(21)	(22)
(1) *Acarmar*		0.003	0.0040	0.004	0.004	0.004	0.004	0.003	0.004	0.003	0.004	0.003	0.003	0.003	0.003	0.004	0.007	0.011	0.003	0.004	0.004	0.003
(2) *Aghimar*	0.03		0.000	0.012	0.005	0.012	0.006	0.012	0.005	0.012	0.006	0.012	0.012	0.012	0.011	0.006	0.006	0.013	0.012	0.007	0.006	0.01
(3) *Agibmar*	0.10	0.08		0.000	0.000	0.000	0.000	0.000	0.000	0.000	0.000	0.000	0.000	0.000	0.000	0.000	0.000	0.000	0.000	0.000	0.000	0.00
(4) *Asenmar*	0.03	0.03	0.10		0.008	0.007	0.008	0.007	0.006	0.007	0.008	0.007	0.007	0.008	0.007	0.008	0.008	0.007	0.007	0.008	0.008	0.01
(5) *Bsaumar*	0.06	0.05	0.08	0.06		0.017	0.020	0.018	0.016	0.017	0.020	0.017	0.017	0.016	0.017	0.020	0.016	0.017	0.017	0.019	0.020	0.02
(6) *Camamar*	0.03	0.03	0.10	0.03	0.06		0.007	0.006	0.007	0.006	0.007	0.006	0.006	0.007	0.006	0.007	0.007	0.006	0.006	0.007	0.007	0.01
(7) *Caubmar*	0.10	0.09	0.00	0.10	0.09	0.10		0.004	0.004	0.004	0.004	0.004	0.004	0.004	0.004	0.003	0.004	0.004	0.004	0.004	0.004	0.00
(8) *Ccrumar*	0.02	0.02	0.09	0.03	0.05	0.02	0.09		0.016	0.008	0.010	0.008	0.007	0.010	0.010	0.010	0.010	0.010	0.010	0.009	0.010	0.01
(9) *Cibemar*	0.06	0.05	0.08	0.06	0.05	0.06	0.08	0.05		0.016	0.017	0.016	0.007	0.016	0.017	0.017	0.015	0.016	0.016	0.016	0.017	0.02
(10) *Fcunmar*	0.02	0.02	0.09	0.02	0.05	0.02	0.09	0.01	0.05		0.009	0.006	0.007	0.009	0.008	0.009	0.008	0.008	0.008	0.008	0.009	0.01
(11) *Gconmar*	0.10	0.08	0.00	0.10	0.08	0.10	0.00	0.09	0.08	0.09		0.000	0.007	0.000	0.000	0.000	0.000	0.000	0.008	0.009	0.009	0.01
(12) *Isubmar*	0.02	0.02	0.09	0.02	0.05	0.02	0.09	0.01	0.05	0.01	0.09		0.007	0.007	0.007	0.008	0.008	0.008	0.007	0.008	0.008	0.01
(13) *Liovmar*	0.01	0.02	0.09	0.02	0.05	0.02	0.09	0.02	0.05	0.02	0.09	0.01		0.007	0.007	0.007	0.007	0.007	0.003	0.004	0.007	0.00
(14) *Lortmar*	0.02	0.04	0.11	0.05	0.07	0.05	0.12	0.04	0.08	0.04	0.11	0.04	0.02		0.000	0.000	0.000	0.000	0.002	0.000	0.000	0.00
(15) *Mboumar*	0.02	0.01	0.08	0.02	0.05	0.02	0.09	0.01	0.05	0.01	0.08	0.01	0.01	0.04		0.006	0.006	0.004	0.005	0.006	0.006	0.01
(16) *Mcromar*	0.10	0.09	0.00	0.10	0.09	0.10	0.01	0.09	0.08	0.09	0.00	0.09	0.09	0.12	0.09		0.004	0.005	0.005	0.004	0.004	0.01
(17) *Mrugmar*	0.08	0.06	0.04	0.08	0.06	0.07	0.04	0.07	0.06	0.07	0.04	0.07	0.07	0.09	0.06	0.04		0.029	0.029	0.025	0.056	0.03
(18) *Msubmar*	0.03	0.02	0.09	0.03	0.05	0.02	0.09	0.02	0.05	0.02	0.09	0.01	0.02	0.04	0.01	0.09	0.07		0.004	0.004	0.004	0.00
(19) *Oclamar*	0.01	0.02	0.09	0.03	0.05	0.03	0.09	0.02	0.06	0.02	0.09	0.02	0.01	0.02	0.02	0.10	0.07	0.02		0.003	0.005	0.00
(20) *Pgramar*	0.07	0.07	0.04	0.08	0.08	0.07	0.04	0.07	0.08	0.07	0.04	0.07	0.06	0.09	0.06	0.04	0.06	0.07	0.07		0.045	0.04
(21) *Rmetmar*	0.10	0.08	0.00	0.10	0.08	0.10	0.00	0.09	0.08	0.09	0.00	0.09	0.09	0.11	0.08	0.00	0.04	0.09	0.09	0.04		0.00
(22) *Tnigmar*	0.01	0.01	0.08	0.02	0.05	0.02	0.08	0.01	0.05	0.01	0.08	0.01	0.01	0.03	0.01	0.09	0.06	0.01	0.01	0.06	0.08	
